# miR-451a Regulates Neuronal Apoptosis by Modulating 14-3-3ζ-JNK Axis upon Flaviviral Infection

**DOI:** 10.1128/msphere.00208-22

**Published:** 2022-06-21

**Authors:** Surajit Chakraborty, Anirban Basu

**Affiliations:** a National Brain Research Centre, Manesar, Haryana, India; Icahn School of Medicine at Mount Sinai

**Keywords:** Cell death, Japanese encephalitis virus, Neuron, West Nile virus, miRNA

## Abstract

Japanese Encephalitis Virus (JEV)/West Nile Virus (WNV)-induced encephalitis, although observed in selective cases, is associated with fatal consequences ranging from decline in cognitive abilities among recovered patients to coma/death. Loss of neuronal cells following viral infection-induced neuronal death imposes significant challenge to the central nervous system (CNS) homeostasis eventually resulting in loss of CNS tissue integrity and poor disease outcome in patients. In our present study, we aim to evaluate the role played by miRNA in modulating neuronal death upon neurotropic flaviviral infections. Infection of neuronal cell line resulted in upregulation of miR-451a abundance. Upon its upregulation, miR-451a has been demonstrated to target 3′-UTR of 14-3-3ζ transcript culminating into downregulation of 14-3-3ζ at the protein level. In response to 14-3-3ζ protein depletion in the cytosol upon flavivirus infection, increased phosphorylation of JNK protein has been shown to take place thus paving way for the cell to undergo apoptosis. Reversal of virus-induced miR-451a-upregulation helped abrogate neuronal apoptosis which is accompanied by a restoration of 14-3-3ζ protein and phosphorylated-JNK abundance to its normal level. Our findings hence provide a possible therapeutic target for preventing JEV/WNV-induced neuronal apoptosis thus improving disease outcome in flaviviral infection-associated encephalitis.

**IMPORTANCE** Neuronal infection by JEV/WNV culminates into neuronal cell death thus contributing to signs and symptoms exhibited by patients that suffer from and that have recovered from JEV/WNV-induced encephalitis. In the present study we have evaluated the role of miRNA in promoting flavivirus-induced neuronal apoptosis. miR-451a has been demonstrated to promote neuronal cell death by targeting 14-3-3ζ protein function. The function of miR-451a in modulating neuronal physiology toward self-destruction has been shown to be independent of its effect upon the virus infection life cycle. The 14-3-3ζ transcript upon being targeted by miR-451a promotes JNK phosphorylation hence culminating into neuronal death by activation of apoptotic machinery. Inhibition of miR-451a upon neuronal infection by JEV/WNV helped reduce apoptotic machinery activation hence providing us with possible future therapeutic strategy in ameliorating flavivirus-induced neurological manifestations and overall disease burden in terms of morbidity.

## INTRODUCTION

Japanese Encephalitis Virus (JEV) belonging to family *Flaviviridae* is a single-stranded positive-sense RNA virus reported to be circulating predominantly across southern and eastern Asia. However, recent reports have demonstrated circulation of JEV at distant locations like Angola (1) and northern Australasia (2) thus pointing toward a far greater geographical range for JEV than considered previously. On the other hand, West Nile Virus (WNV) belonging to family *Flaviviridae* has been documented to exhibit a wide range of geographical distribution including Australia, Asia, Africa, the middle east, and America (3). Spread by mosquitoes of *Culex* species, JEV in endemic areas predominantly affect children. However, nonimmune adults that have never experienced JEV infection thus possessing no JEV-specific immune memory, when introduced into the endemic population, also exhibit new cases of JEV infection (4). Similarly, introduction of JEV into non-JEV-endemic area leads to outbreak characterized by prevalence of JEV cases in both children and adults (5). In addition to a very comprehensive and systematic study (6) demonstrating 69,000 annual incidences of JEV infection across the last decade, observations reported by Mathers et al. (7), denoting the severe global burden imposed by JEV infection in the form of 709,000 disability-adjusted life years annually, indicates high magnitude of burden imposed by JEV infection thus warranting further research aimed at ameliorating JEV-associated morbidities. While both JEV and WNV are being transmitted by *Culex* species of mosquitoes, unlike JEV, which possesses a predilection for affecting young children, WNV affects patients of all ages (8). Infection by JEV or WNV is characterized by a range of overlapping symptoms varying from undifferentiated febrile illness, headache, vomiting, coryza, and diarrhea to CNS involvement characterized by loss of consciousness, seizures, encephalitis, and coma culminating into death (8). Multiple studies (9–11) reporting pathological findings regarding the CNS involvement upon JEV infection from autopsy of CNS tissues from deceased patients indicate diffuse as well as focal CNS damage concomitant with distribution of viral antigen-positive neurons across the thalamus, midbrain, hippocampus, and temporal cortex in JEV and brainstem and spinal cord in case of WNV. Involvement of basal ganglia observed in brain sections (9) from deceased patients are consistent with the parkinsonian defects exhibited by patients suffering from JEV-induced encephalitis. These studies in addition to reports (12, 13) demonstrating histological findings of CNS involvement in macaques upon JEV infection underscore the significance of neuronal damage that manifests into a plethora of neurological symptoms exhibited by patients and thus further warrants investigation aimed at elucidating mechanistic basis of neuronal death upon JEV/WNV invasion into the CNS.

Both exaggerated proinflammatory response and neuronal infection by JEV have been reported to cause neuronal death via activation of apoptotic machinery. Findings from multiple reports (14–19) offer insights into mechanistic bases for immune response-mediated neuronal damage upon JEV/WNV infection, implicating dysregulated action of both innate and adaptive arms of immunity. Overactivation of innate immune cells like macrophages and microglia, and amplified production and action of soluble factors like IL-6, TNF-α, acting in concert with dysregulated CD4^+^T- and CD8^+^T cells, in case of JEV and WNV respectively, result in irreversible damage to neurons. On the other hand, molecular basis for JEV infection-induced cellular damage independent of adverse effects of immune response has been investigated upon by multiple groups (19–23). JEV has been observed to activate various arms of the unfolded protein response (UPR) pathway, which contribute to the process of cellular death upon irreparable damages imposed by JEV infection. Downstream signaling pathways initiated from MAP Kinase JNK have also been shown to promote cellular apoptosis upon JEV/WNV infection by loss-of-function causation-testing experiments.

miRNAs being well studied for their fine-tuning/modulating effect upon diverse cellular physiological and pathological processes (24) play a vital role in various aspects of host response upon viral infections(25). Our present study hence aims to examine the role of miRNA miR-451a in flavivirus-induced neuronal apoptosis in the absence of any effect imparted by exacerbated proinflammatory response. This study provides an outline of the molecular pathway leading from miR451a upregulation to neuronal death via reduction of cellular abundance of 14-3-3ζ protein, which in turn leads to enhanced phosphorylation of JNK. Experimental evidence provided herein supporting the role of miR-451a in promoting neuronal apoptosis in a cell-autonomous fashion provides us with a possible therapeutic target that can be utilized to abrogate JEV/WNV infection-associated neuronal death and ameliorate the debilitating neurological sequalae prevalent among recovered patients.

## RESULTS

### Overexpression of miR-451a in neuronal cells upon infection by JEV.

**FIG 1:**
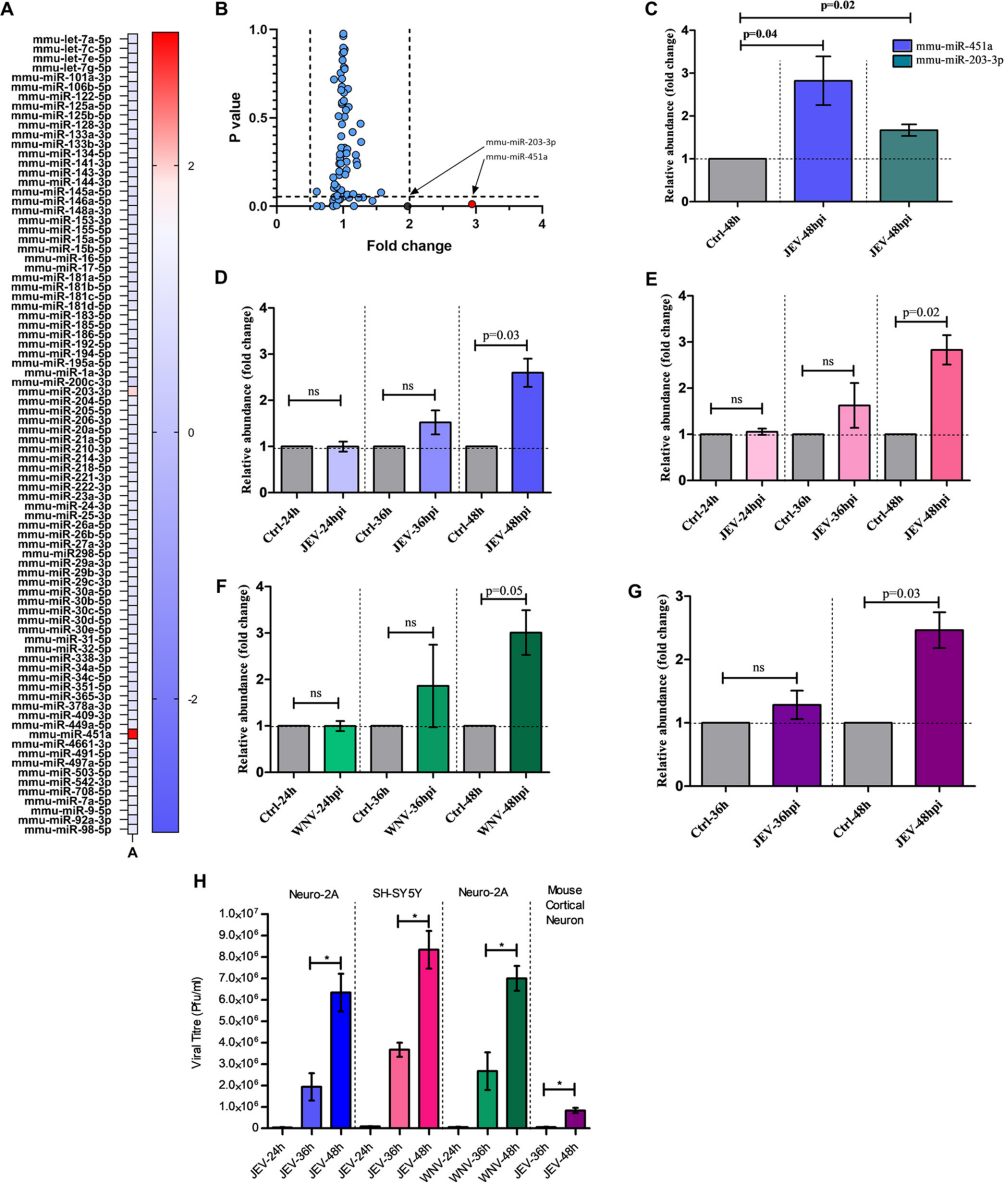
Neuronal infection by flaviviruses induces upregulation of miR-451a. (A) RNA isolated from JEV-infected Neuro-2a cells at an MOI of 3 for 48 h were subjected to miRNA-qRT-PCR array; the analyzed data have been presented as a heatmap representing fold change obtained for each miRNA. (B) Data from the miRNA-qRT-PCR array plotted with fold change and respective *P*-values in different axes denoting statistical significance of miR-451a and miR-203-3p upregulation upon JEV infection. (C) miRNA qRT-PCR microarray findings were validated in Neuro-2A cells using qRT-PCR assessing miR-451a abundance in comparison to that of uninfected cells. Time-dependent expression profile of miR-451a upon JEV infection of Neuro-2A cells (D) and SH-SY5Y cells (E) and WNV infection of Neuro-2A cells (F) at an MOI of 3 were evaluated, employing qRT-PCR in comparison to uninfected cells maintained for respective time points. RNA isolated from mouse primary cortical neurons (G) infected by JEV for 36 and 48 h was subjected to qRT-PCR analysis for studying expression kinetics of miR-451a. Each of the qRT-PCR data analyzing miR-451a expression was normalized using SNORD68 snRNA abundance. (H) Supernatant collected from JEV/WNV-infected Neuro-2A, JEV-infected SH-SY5Y, and JEV-infected mouse cortical neuronal culture were collected and subjected to plaque assay for detection of viral growth kinetics. Bar graphs shown in the figure represent data from three biological replicates in the form of mean ± SD. Comparisons between uninfected and respective infected samples were performed using Student's *t* test with individual *P*-values indicated in the figures. *, *P* < 0.05, ns= non-significant.

In an attempt to evaluate the differentially regulated apoptotic-miRNAs upon infection by JEV, RNA isolated from JEV-infected Neuro-2A cells at multiplicity-of-infection 3 (MOI) for 48-h was subjected to miRNA-PCR microarray using MiScript miRNA PCR array, mouse, apoptosis (Qiagen product number: 331221, catalogue number: MIMM-114ZA) (Fig. 1A). The heatmap shown in Fig. 1A and fold changes shown in Fig. 1B upon JEV infection of each miRNA against respective *P*-values shows miR-451a and miR-203-3p as significantly upregulated miRNAs. Out of these two differentially upregulated miRNAs, miR-451a was observed to be the most severely upregulated candidate (miR-451a: 2.94-fold vs. miR-203-3p: 1.971-fold) along with significant statistical difference, warranting investigation into its role in virus-induced neuronal apoptosis. Raw data obtained from miRNA-PCR array were analyzed using Qiagen data analysis software available online and have been provided as Table S1. Validation of miRNA PCR-microarray findings utilizing qRT-PCR analysis of RNA samples from JEV-infected Neuro-2A cells for mature-miR-451a and mature-miR-203-3p expression indicated significant upregulation in their abundance at 48 h postinfection (hpi) (Fig. 1C). A time-dependent study aimed at deciphering kinetics of miR-451a expression upon JEV infection revealed an increase in miR-451a abundance at 48 hpi but not at 24 & 36 hpi in both mouse and human neuronal cell-line Neuro-2A (Fig. 1D) and SH-SY5Y (Fig. 1E), respectively. miR-451a abundance was also demonstrated to be increased in mouse primary cortical neuronal cells at 48 hpi by JEV (Fig. 1G). In order to check whether miR-451a upregulation upon infection by JEV is virus-specific or applicable to any other virus belonging to family *Flaviviridae*, we assessed miR-451a expression status following infection of Neuro-2A cells by WNV and observed a similar increase in miR-451a expression level at 48 hpi but not prior to that (Fig. 1F). Viral growth kinetics data of JEV in Neuro-2A, mouse cortical neurons, SH-SY5Y, and WNV in Neuro-2A (Fig. 1H) show productive infection increasing in magnitude from 36 hpi to 48 hpi as assessed by plaque assay. The delay in onset of productive infection and miR-451a upregulation might be attributed by the time taken by the virus to activate respective host molecular circuitry, resulting in initiation of miR-451a transcription, hence miR-451a upregulation. Infection of SH-SY5Y cells by WNV also exhibited increased upregulation of miR-451a at 48 hpi (Fig. S5A) but not following infection for 24 and 36 h. Analysis of JEV-infected autopsy brain section exhibited enhanced miR-451a abundance when compared to the control (Fig. S1A). Similar trend of increment in miR-451a abundance was demonstrated by brain samples of JEV- and WNV-infected BALB/c mouse model at late-stage of infections (Fig. S1B and S1C, respectively). Collectively, this evidence points toward JEV/WNV infection resulting in increased miR-451a abundance in neurons/neural tissue.

10.1128/msphere.00208-22.1 TABLE S1 Expression data of miRNAs of JEV-infected Neuro-2A cells upon infection for 48 h and their respective statistical significance. Download Table S1, PDF file, 0.04 MB. Copyright © 2022 Chakraborty and Basu. 2022 Chakraborty and Basu. https://creativecommons.org/licenses/by/4.0/ This content is distributed under the terms of the Creative Commons Attribution 4.0 International license.

10.1128/msphere.00208-22.2 FIG S1 miR-451a is upregulated in JEV-infected human autopsy brain-tissue and JEV/WNV-infected mouse brain-tissue. (A) RNA isolated from uninfected and JEV-infected human autopsy brain sections was subjected to qRT-PCR analysis for evaluating miR-451a abundance. qRT-PCR data corresponding to miR-451a abundance was normalized using SNORD68 snRNA. The bar graphs denoted in figure represent data from three different brains per group. miR-451a expression was assessed in JEV- (B)/WNV-infected mouse brain sample (C) in comparison to uninfected mouse brain employing qRT-PCR. Mouse brain tissue miR-451a expression data was normalized using SNORD68 snRNA abundance. Bars indicate data collected from 5 animals in each group. All the data in the figure have been presented as mean ± SD. Statistical significance of differences in miR-451a expression between 2 groups has been calculated using Student’s *t*-test. Download FIG S1, TIF file, 0.3 MB. Copyright © 2022 Chakraborty and Basu. 2022 Chakraborty and Basu. https://creativecommons.org/licenses/by/4.0/ This content is distributed under the terms of the Creative Commons Attribution 4.0 International license.

10.1128/msphere.00208-22.6 FIG S5 Role of miR-451a in modulating SH-SY5Y cell death upon infection by West Nile Virus. (A) RNA isolated from WNV-infected SH-SY5Y cells were subjected to quantitative PCR for determination of time-dependent expression profile of mature miR-451a using SNORD68 as normalization control. (B) Protein isolated from WNV-infected (48 h) SH-SY5Y cells transfected with miR-451a inhibitor/inhibitor-control was subjected to immunoblotting analysis for determination of relative abundance of total- and cleaved-caspase-3. The bar graph indicates densitometric analysis of c-caspase-3-fold change (normalized using total-caspase-3). (C) Protein isolated from WNV-infected (48 h) SH-SY5Y cells transfected with miR-451a inhibitor/inhibitor-control/miR-451a mimic/mimic-control were used for immunoblot analysis of WNV-NS3 protein abundance. Supernatant collected from WNV-infected (48 h) SH-SY5Y cells transfected with miR-451a inhibitor/inhibitor-control (D) and miR-451a inhibitor/inhibitor-control (E) was used to measure WNV titer (PFU/mL) employing plaque assay. (F) Protein isolated from WNV-infected SH-SY5Y cells for time periods as indicated in the figure was subjected to immunoblotting to assess the time-dependent expression profile of p-IRE1α, total-IRE1α, p-PERK, total-PERK, ATF-4, CHOP, p-JNK, total-JNK. ß-actin was used as a loading control. The bar graph indicates quantitative analysis of p-JNK abundance at multiple time points following infection by WNV and normalized using t-JNK expression. (G) p-JNK expression was evaluated using protein isolated from WNV-infected (48 h) SH-SY5Y cells transfected with miR-451a inhibitor/inhibitor-control/miR-451a mimic and mimic-control. The bar graph indicates quantitative analysis of p-JNK abundance under different conditions and were normalized using t-JNK abundance. (H) Protein isolated from WNV-infected SH-SY5Y cells for time periods as denoted in figure was subjected to immunoblotting to assess the time-dependent expression profile of 14-3-3ζ. The bar graph denotes quantitative analysis of expression kinetics of 14-3-3ζ (normalized to ß-actin) immunoblots following SH-SY5Y infection by WNV. (I) Protein harvested from WNV-infected (48 h) SH-SY5Y cells transfected with miR-451a inhibitor/inhibitor-control/miR-451a mimic and mimic-control prior to infection were subjected to western blotting to assess 14-3-3ζ expression. The bar graph indicate densitometric analysis of 14-3-3 ζ abundance (normalized to ß-actin) as denoted by immunoblotting experiments upon miR-451a inhibitor/inhibitor-control/miR-451a mimic and mimic-control transfection. All the blots presented here represent data from 3 independent experiments with similar outcomes. ß-actin served as a loading control. Bar graphs denote data from three independent experiments in the form of mean ± SD. **P < 0.001, *P < 0.05, by two-tailed student’s t-test. Download FIG S5, TIF file, 1.5 MB. Copyright © 2022 Chakraborty and Basu. 2022 Chakraborty and Basu. https://creativecommons.org/licenses/by/4.0/ This content is distributed under the terms of the Creative Commons Attribution 4.0 International license.

### miR-451a regulates JEV/WNV-induced neuronal apoptosis.

**FIG 2:**
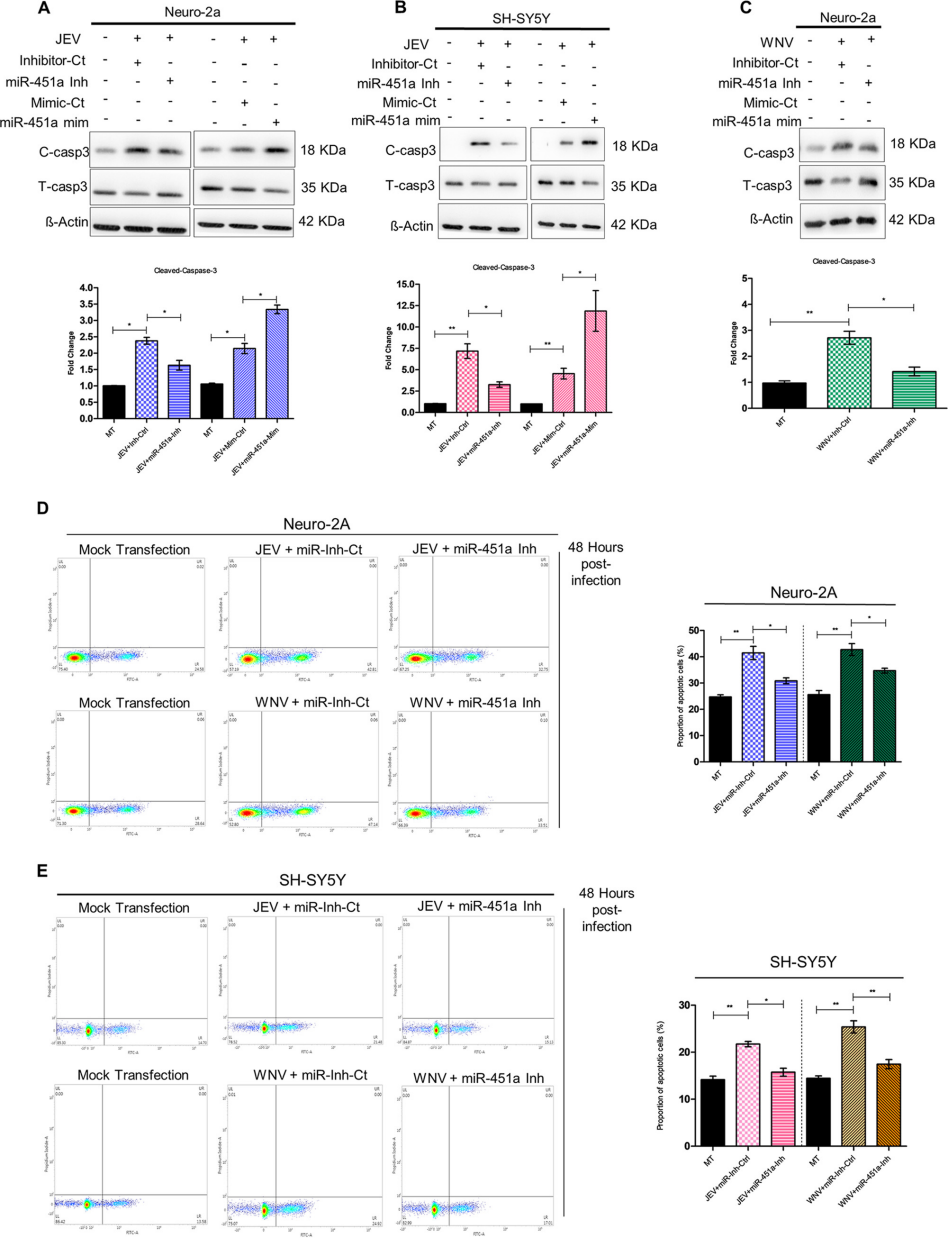
miR-451a promotes JEV/WNV-induced neuronal apoptosis. Following 24 h of transfection with miR-451a inhibitor (miR-451a-Inh)/miR-451a mimic (miR-451a-mim)/miR inhibitor-control (Inhibitor-Ct)/mimic-control (Mimic-Ct) as indicated in the figure, Neuro-2A (A) and SH-SY5Y (B) cells were infected with JEV for 48 h and WNV (C) for 48 h at MOI 3. Protein isolated from these samples was subjected to immunoblotting analysis for estimation of total- and cleaved-caspase-3 abundance. All the blots presented in the figure represent data from three independent experiments with similar results. ß-actin was used as a loading-control. Molecular weight of protein molecules in Kilodalton (kDa) has been stated beside the respective immunoblots. Densitometric analysis of immunoblots demonstrating effect of miR-451-a inhibitor/inhibitor-control/miR-451a mimic/mimic-control transfection in JEV-infected (48 h) Neuro-2A (A, bottom: ratio of cleaved-caspase-3 to total-caspase-3), JEV-infected SH-SY5Y (B, bottom: ratio of cleaved-caspase-3 to total-caspase-3) and WNV-infected Neuro-2A cells (C, bottom: ratio of cleaved-caspase-3 to total-caspase-3) upon cleaved-caspase-3 normalized to total-capase-3. JEV/WNV-infected Neuro-2A (D) and SH-SY5Y (E) cells transfected with miR-451a inhibitor/Inhibitor-Ct prior to infection were collected after indicated time period and stained using annexin-FITC and propidium iodide followed by analysis using BDFacs Verse for detection of proportion of apoptotic cells along with their quantitative estimation as bar graphs representing proportion of apoptotic cells (D, right and E, right). Bar graphs in the form of mean ± SD represent data from three independent experiments. *, *P* < 0.05, **, *P* < 0.01, by two-tailed Student’s *t* test.

In order to assess role of miR-451a in modulating neuronal apoptosis upon JEV/WNV infection, we transfected cells with miR-451a inhibitor or mimic (chemically synthesized double stranded RNA molecule mimicking endogenous mature miR-451a) for 24 h prior to infection for another 48 h. In both mouse and human neuronal cell-lines Neuro-2A and SH-SY5Y, miR-451a inhibitor and mimic were found to inhibit and enhance JEV/WNV-induced miR-451a upregulation, respectively (Fig. S2A–H). Inhibition of JEV/WNV-induced miR-451a upregulation in Neuro-2A/SH-SY5Y cells resulted in reduced activation of apoptotic pathway as indicated by reduced cleavage of caspase-3 at 48 hpi with respect to inhibitor-control-transfected virus-infected samples (Fig. 2A– 2C, and S5B). In accordance with these observations, overexpression of miR-451a in the face of JEV infection in Neuro-2A/SH-SY5Y cells enhancing miR-451a abundance led to enhanced apoptotic signaling as exemplified by reinforcement of caspase-3 cleavage (Fig. 2A, B). In addition to cleaved-caspase-3/total-caspase-3 ratio, annexin-propidium iodide staining of JEV/WNV-infected Neuro-2A/SH-SY5Y cells transfected with miR-451a inhibitor in the context of virus-induced miR-451a upregulation showed reduction (modest but statistically significant) in percentage of apoptotic cells when compared to infected cells transfected with inhibitor-control (Fig. 2D and 2E). Moreover, TUNEL assay of JEV/WNV-infected Neuro-2A/SH-SY5Y cells transfected with miR-451a inhibitor exhibited reduced apoptosis as exemplified by reduced incorporation of fluorescently tagged deoxy uridine nucleotide with respect to infected cells transfected with inhibitor-control (Fig. S4A, S4B). Supported by the aforementioned evidence, miR-451a upregulation in neuronal cells upon JEV/WNV-infection hence can be concluded to promote neuronal demise via activation of apoptotic program.

10.1128/msphere.00208-22.3 FIG S2 miR-451a-inhibitor and miR-451a mimic restores and upregulates miR-451a abundance respectively in JEV/WNV-infected Neuro-2A and JEV/WNV-infected SH-SY5Y cells. Relative abundance of miR-451a was determined in miR-451a-inh/Inhibitor-Ctrl (A) or miR-451a-mim/Mimic-Ctrl-transfected (B) JEV-infected Neuro-2A cells (for 48-hours), JEV-infected (for 48-hours) SH-SY5Y cells transfected with miR-451a inh/Inhibitor-Ctrl (C) or miR-451a-mim/Mimic-Ctrl (D), WNV-infected (48 h) Neuro-2A cells transfected with miR-451a inh/Inhibitor-Ctrl (E) or miR-451a-mim/Mimic-Ctrl (F) and WNV-infected (for 48-hours) SH-SY5Y cells transfected with miR-451a inh/Inhibitor-Ctrl (G) or miR-451a mim/Mimic-Ctrl (H) using qRT-PCR. Bar graphs shown in the figure represent data as mean ± SD from 3 biological replicates. Statistical significance of differences between miR-451a relative abundance belonging to 2 different experimental conditions has been calculated using Student’s t-test. Relative expression of miR-451a has been obtained using SNORD68 snRNA abundance as normalizing-control. *P < 0.05, by two-tailed Student’s *t*-test. Download FIG S2, TIF file, 1.3 MB. Copyright © 2022 Chakraborty and Basu. 2022 Chakraborty and Basu. https://creativecommons.org/licenses/by/4.0/ This content is distributed under the terms of the Creative Commons Attribution 4.0 International license.

10.1128/msphere.00208-22.5 FIG S4 miR-451a-inhibitor upon transfection successfully inhibits neuronal cell death upon JEV/WNV infection in Neuro-2A and SH-SY5Y cells. Neuro-2A and SH-SY5Y cells transfected with miR-451a inh/Inhibitor-Ctrl followed by JEV (A) or WNV (B) infection for 48 h were subjected to labeling with FITC-tagged deoxy uridine and observed under fluorescence microscope for evaluation of deoxy uridine incorporation indicating cell death. Scale bar indicated in micrographs measures 100 μm; original magnification, ×10. Download FIG S4, TIF file, 1.5 MB. Copyright © 2022 Chakraborty and Basu. 2022 Chakraborty and Basu. https://creativecommons.org/licenses/by/4.0/ This content is distributed under the terms of the Creative Commons Attribution 4.0 International license.

### miR-451a-mediated neuronal death upon JEV/WNV infection is independent of its effect upon viral propagation.

**FIG 3:**
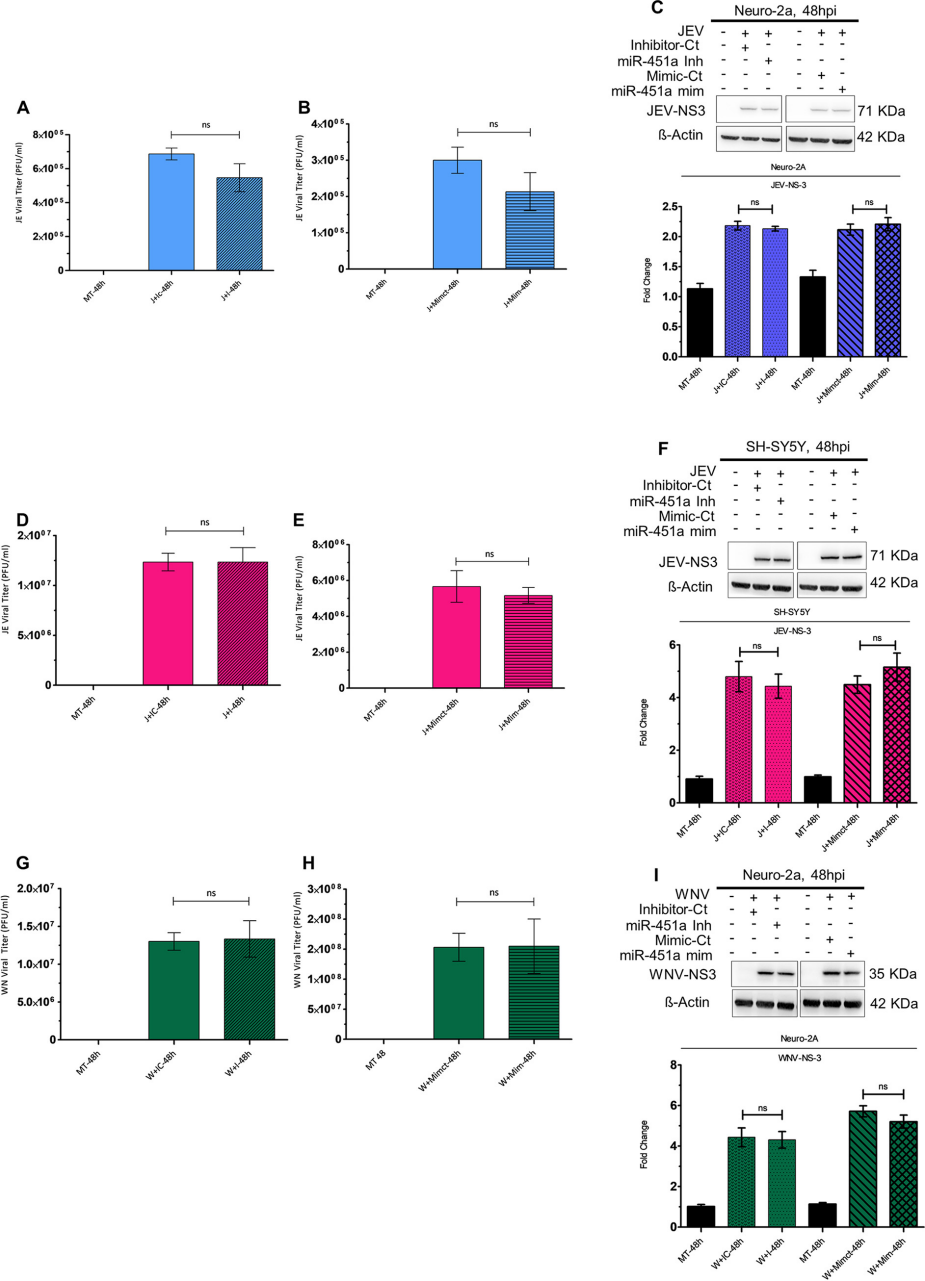
miR-451a upregulation modulates neuronal apoptosis without affecting JEV/WNV propagation. Supernatant obtained from Neuro-2A transfected with miR-451a inhibitor/inhibitor-Ct (A) and miR-451a mimic/Mimic-Ct (B), and SH-SY5Y transfected with miR-451a-inhibitor/inhibitor-Ct (D) and miR-451a mimic/Mimic-Ct (E) for 24 h prior to infection by JEV (MOI 3) for another 48 h was subjected to plaque assay for determination of viral titer. Similarly, cell-culture media collected from Neuro-2A transfected with miR-451a inhibitor/inhibitor-Ct (G) and miR-451a mimic/Mimic-Ct (H) for 24 h followed by 48 h of WNV (MOI 3) infection were investigated for viral titer estimation utilizing plaque assay. Protein isolated from JEV- (C) and WNV-infected (I) Neuro-2A-cells and JEV-infected SH-SY5Y-cells (F) transfected with miR-451a inhibitor/Inhibitor-Ct and miR-451a mimic/Mimic-Ct, as indicated in the figure, was subjected to immunoblot analysis to determine abundance of JEV- and WNV-NS3 protein expression. The blots shown in the figure represent data from 3 independent experiments with similar results. ß-actin served as a loading-control. Stated beside each immunoblot is the molecular weight in kDa of the respective probed molecule. Quantitative analysis of immunoblots demonstrate the effect of miR-451-a inhibitor/inhibitor-control/miR-451a mimic/mimic-control transfection in JEV-infected Neuro-2A cells (C, bottom: ratio of JEV-NS3 to ß-actin), JEV-infected SH-SY5Y cells (F, bottom: ratio of JEV-NS3 to ß-actin), and WNV-infected Neuro-2A cells (I, bottom: ratio of WNV-NS3 to ß-actin) upon viral NS-3 protein abundance (normalized to ß-actin). Data in bar graphs are represented as mean ± SD from three experimental replicates with similar outcomes. Statistical significance of differences between data belonging to 2 different experimental conditions was determined using Student's *t* test. ns= non-significant, using two-tailed Student's *t* test.

To evaluate whether miR-451a’s effect upon neuronal apoptosis is dependent upon any changes in magnitude of viral propagation, we measured titers of JEV and WNV released by Neuro-2A and SH-SY5Y cells 48 hpi in the presence of miR-451a inhibitor/mimic or miR-inhibitor-control/miR-mimic-control. JEV-infected Neuro-2A cells transfected with miR-451a inhibitor exhibited no difference in virus released into supernatant with respect to infected-cells transfected with miR-inhibitor-control (Fig. 3A). Similarly, overexpression of miR-451a in the context of JEV infection in Neuro-2A cells did not result in any differences in JEV titer (Fig. 3B). Estimation of JEV nonstructural (NS)-protein NS-3 abundance upon both inhibition and overexpression of miR-451a failed to generate any differences with respect to JEV-infected Neuro-2A cells transfected with miR-inhibitor-control/miR-mimic-control (Fig. 3C). Modulation of JEV-induced miR-451a upregulation in JEV-infected SH-SY5Y cells by employing miR-451a inhibitor/mimic also resulted in no significant changes in JEV titer or NS-3 abundance (Fig. 3D to F). Similar to findings observed in the case of Neuro-2A infection by JEV, inhibition/overexpression of miR-451a in the context of WNV infection did not exhibit any alterations in WNV propagation either at the level of released virus or at the level of viral protein translation (Fig. 3G–3I). WNV-infected SH-SY5Y cells transfected with miR-451a inhibitor or mimic displayed no difference in WNV NS-3 protein abundance (Fig. S5C) and WNV release in supernatant as shown by plaque assay (Fig. S5D, S5E) when compared with WNV-infected SH-SY5Y cells transfected with inhibitor-control or mimic-control, respectively.

### JEV/WNV-induced miR-451a upregulation promotes phosphorylation of JNK.

**FIG 4:**
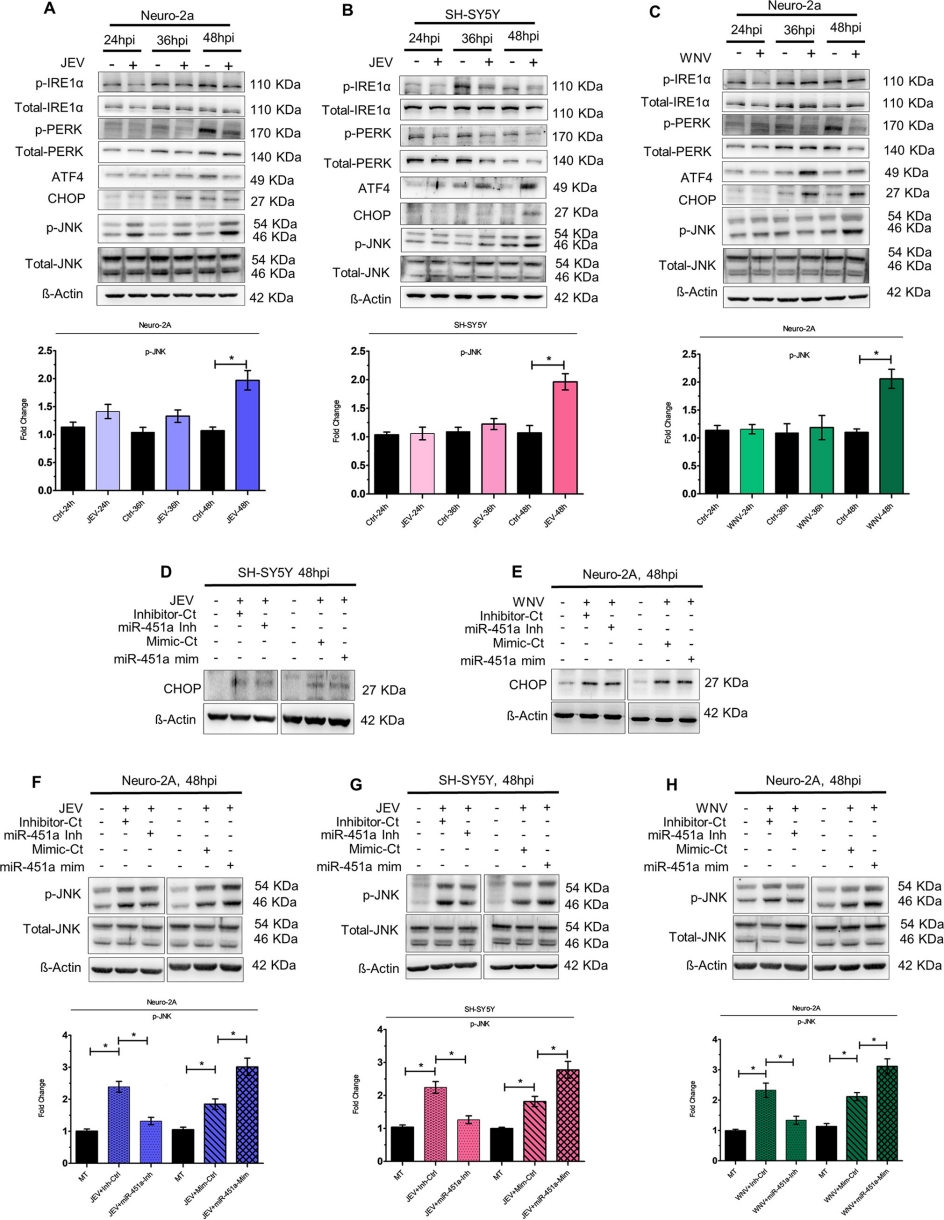
miR-451a modulates JNK phosphorylation in the context of JEV/WNV infection of neuronal cells. Protein isolated from JEV-infected (MOI 3) Neuro-2A cells (A)/SH-SY5Y cells (B) and WNV-infected (MOI 3) Neuro-2A cells (C) cells for different time-periods as indicated in the figure was investigated for time-dependent expression profile of p-IRE1α, total-IRE1α, p-PERK, total-PERK, ATF-4, CHOP, p-JNK, and total-JNK. Densitometric analysis of immunoblots demonstrate time-dependent kinetics of p-JNK (normalized to total-JNK) in JEV-infected Neuro-2A (A, bottom: ratio of phospho-JNK to total-JNK), JEV-infected SH-SY5Y cells (B, bottom: ratio of phospho-JNK to total-JNK), and WNV-infected Neuro-2A cells (C, bottom: ratio of phospho-JNK to total-JNK). CHOP abundance was evaluated in JEV-infected SH-SY5Y cells (D) or WNV-infected Neuro-2A-cells (E) transfected with miR-451a inhibitor/inhibitor-control or miR-451a-mimic/mimic-control as denoted in the figure. Similarly, p-JNK and total-JNK expression was studied utilizing proteins isolated from JEV-infected (F) and WNV-infected (H) Neuro-2A cells and JEV-infected SH-SY5Y cells (G) transfected with miR-451a inhibitor/inhibitor-control, and miR-451a mimic/mimic-control. All the immunoblots presented in the figure represent data from experimental triplicate with similar results. ß-actin has been used as a loading-control. Molecular weight of each probed molecule has been stated in kDa beside the corresponding immunoblots. Quantitative analysis of immunoblots demonstrating effect of miR-451a inhibitor/inhibitor-control/miR-451a mimic/mimic-control transfection in JEV-infected Neuro-2A (F, bottom: ratio of phospho-JNK to total-JNK), JEV-infected SH-SY5Y (G, bottom: ratio of phospho-JNK to total-JNK), and WNV-infected Neuro-2A (H, bottom: ratio of phospho-JNK to total-JNK) upon p-JNK abundance (normalized to total-JNK). Bar graphs represent data in the form of mean ± SD from three independent experiments with similar outcomes. *, *P* < 0.05, by two-tailed Student's *t* test.

Multiple documented reports provide ample evidence supporting the role of endoplasmic reticulum (ER) stress in virus-induced cell death (26). Flaviviruses like JEV and WNV, upon infection have been demonstrated to activate unfolded protein response (UPR), which in turn plays a vital role in cellular apoptosis (27, 28). Since under our experimental conditions, miR-451a was observed to be upregulated at 48 hpi, we assessed the time-dependent expression profile of a battery of signaling molecules belonging/downstream to multiple arms of ER stress pathway to check which of them is being found to be dysregulated at 48 hpi. ER stress pathway molecules attributing to flavivirus-induced cell death according to earlier reports and demonstrating altered expression profile in neuronal cells at 48 hpi might be modulated by miR-451a upregulation, thus promoting neuronal apoptosis. We studied expression profiles of molecules like phosphorylated-IRE1-α (p-IRE1-α), total-IRE1-α phosphorylated-PERK (p-PERK), total-PERK, ATF-4, CHOP and phosphorylated-JNK (p-JNK), total-JNK upon infection by JEV and WNV of Neuro-2A (Fig. 4A and 4C) and SH-SY5Y cells (Fig. 4B, S5F). Of the above-mentioned molecules, CHOP abundance was found to be upregulated at 48 hpi in JEV-infected SH-SY5Y cells (Fig. 4B) and WNV-infected Neuro-2A cells (Fig. 4C). However, modulation of miR-451a using miR-451a inhibitor or miR-451a mimic did not result in any change in CHOP expression in JEV-infected SH-SY5Y (Fig. 4D) and WNV-infected n-2A cells (Fig. 4E), thus ruling out the possibility that miR-451a promotes apoptosis upon infection in a CHOP-dependent fashion. Upregulation of p-JNK abundance in JEV-infected Neuro-2A cells (Fig. 4A), WNV-infected Neuro-2A cells (Fig. 4C), as well as JEV-infected (Fig. 4B) and WNV-infected SH-SY5Y cells (Fig. S5F) at 48 hpi were found to be inhibited or enhanced upon miR-451a inhibition or overexpression, respectively (Fig. 4F–4H, and S5G), thus emphasizing the role of miR-451a in modulating phosphorylation of JNK.

### 14-3-3ζ protein acts as a target for miR-451a in neuronal cells.

**FIG 5:**
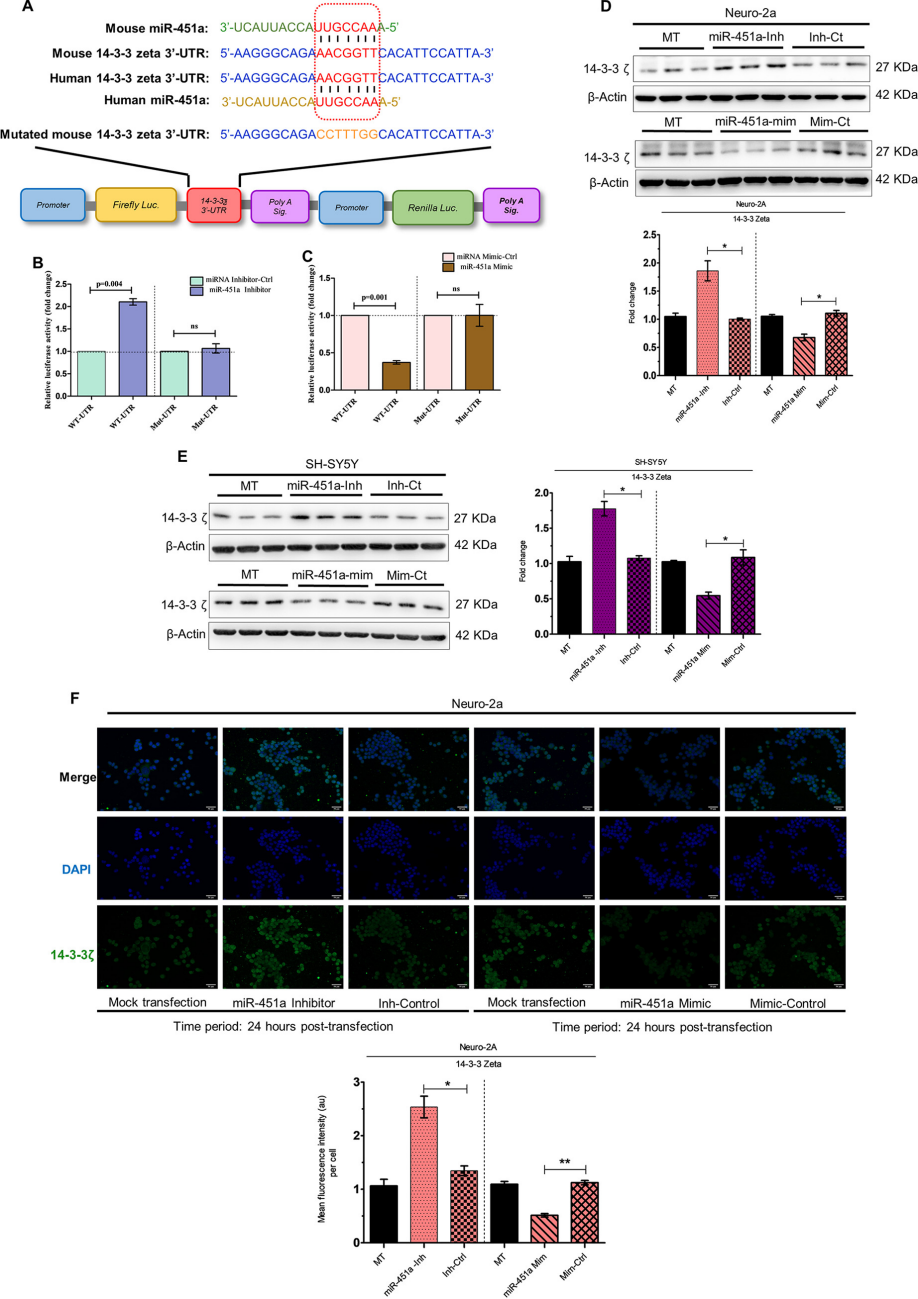
14-3-3ζ acts as a potential target for miR-451a in neuronal cells. Mouse and human miR-451a sequence and their respective binding sites at 3′-UTR of 14-3-3ζ (WT-UTR) are demonstrated (A). The miR-451a binding site in 14-3-3ζ 3′-UTR is marked in red. The mouse mutant 3′-UTR (Mut-UTR), which possesses mutations in the miR-451a binding-site is marked in orange. Dual luciferase assay employing transfection of Neuro-2A cells by WT-UTR or Mut-UTR along with miR-451a inhibitor/inhibitor-control (B) or miR-451a mimic/mimic-control (C) was performed, and data are presented as relative luciferase activity (RLA). Bar graphs demonstrating RLA in the form of mean ± SD were generated using data from 3 independent transfection experiments. Firefly luciferase activity was normalized using renilla luciferase activity expressed by the same reporter plasmid. Statistical significance of differences in RLA were estimated using Student's *t* test. Proteins isolated from mock-transfected (MT), miR-451a inhibitor, inhibitor-control-, miR-451a mimic-, and mimic-control-transfected Neuro-2A (D) or SH-SY5Y (E) cells for 24 h were subjected to immunoblotting experiments for analyzing 14-3-3ζ abundance. Blots are representative of experimental triplicate with similar outcome. ß-actin served as a loading control. Densitometric analysis of 14-3-3ζ abundance (normalized to ß-actin) in Neuro-2A (D, bottom: ratio of 14-3-3ζ to ß-actin) and SH-SY5Y cells (E, right: ratio of 14-3-3ζ to ß-actin) upon transfection with miR-451a-inhibitor/inhibitor-control or miR-451a mimic/mimic-control. ß-actin was used as a loading control. Immunocytochemical analysis of 14-3-3ζ expression following mock-, miR-451a inhibitor-, inhibitor-control-, miR-451a mimic-, and mimic-control-transfected Neuro-2A cells (F) for 24 h. Scale bar denoted in each micrograph measures 50 μm; original (×20 magnification). Quantitative analysis of effects of miR-451a-inhibitor/inhibitor-control or miR-451a mimic/mimic-control upon relative abundance of 14-3-3ζ in Neuro-2A cells (F, bottom). Data presented in bar graphs in the form of mean ± SD denote data collected from three independent experiments with similar outcomes. *, *P* < 0.05; **, *P* < 0.01; ns=non-significant, by two-tailed Student's *t* test.

Employing the TargetScan website, we observed both mouse and human 14-3-3ζ gene transcripts (also known as YWHAZ) as a potential target for mouse and human miR-451a, respectively, as denoted by complementarity between mouse and human miR-451a seed sequence and 3′-UTR of mouse and human 14-3-3ζ mRNA (Fig. 5A). When subjected to dual luciferase assay, mouse miR-451a inhibitor or mimic transfection resulted in enhancement (Fig. 5B) or abrogation (Fig. 5C) of luciferase activity, respectively, when compared to transfection with miR-inhibitor-control or miR-mimic-control, respectively. The above-mentioned changes in luciferase activity were demonstrated to be abolished upon mutation of mouse miR-451a-binding site in 3′-UTR of 14-3-3ζ transcript (Mut-UTR) (Fig. 5B and C). Transfection of uninfected neuronal cell lines Neuro-2A (Fig. 5D) or SH-SY5Y (Fig. 5E) with miR-451 inhibitor or mimic culminated into an increase or decrease in 14-3-3ζ protein abundance, respectively, with respect to transfection by miR-inhibitor-control/miR-mimic-control, pointing toward the role of 14-3-3ζ transcript acting as a potential target for miR-451a in Neuro-2A and SH-SY5Y. This modulation of 14-3-3ζ was accompanied by a concomitant decrease and increase in miR-451a relative abundance, respectively (Fig. S3A and S3B), indicating potent transfection by miR-451a inhibitor/mimic. The observations presented in Fig. 5D and 5E were reconfirmed by immunocytochemical analysis of 14-3-3ζ abundance in Neuro-2a cells upon transfection with miR-451a inhibitor/mimic (Fig. 5F). Cells transfected with miR-451a inhibitor or mimic exhibited increased or decreased 14-3-3ζ protein abundance when compared to cells transfected with miR-inhibitor-control or miR-mimic-control respectively. In addition to the evidence pointing toward the role of 14-3-3ζ transcript acting as a potential target for miR-451a, an earlier study reporting role of 14-3-3ζ in blocking basal level of JNK phosphorylation(29) named 14-3-3ζ as the most-probable candidate for investigation regarding its involvement in virus-induced miR-451a upregulation-associated neuronal apoptosis.

10.1128/msphere.00208-22.4 FIG S3 miR-451a-inhibitor and miR-451a-mimic upon transfection successfully inhibits and overexpresses miR-451a respectively in neuronal cells. RNA isolated from (A) Neuro-2A and (B) SH-SY5Y cells upon mock-/miR-451a-inh-/Inhibitor-Ctrl- and mock-/miR-451a mim-/Mimic-Ctrl-transfection for 24 h was subjected to qRT-PCR for evaluating miR-451a relative abundance (normalized using SNORD68 snRNA abundance). Figures presented as bar graphs represent data in the form of mean ± SD collected from 3 independent experiments. Statistical significance of differences in miR-451a expression between 2 different conditions has been calculated employing Student’s *t*-test. Download FIG S3, TIF file, 0.8 MB. Copyright © 2022 Chakraborty and Basu. 2022 Chakraborty and Basu. https://creativecommons.org/licenses/by/4.0/ This content is distributed under the terms of the Creative Commons Attribution 4.0 International license.

### JEV/WNV-infection downregulates 14-3-3ζ protein abundance in neuronal cells.

**FIG 6:**
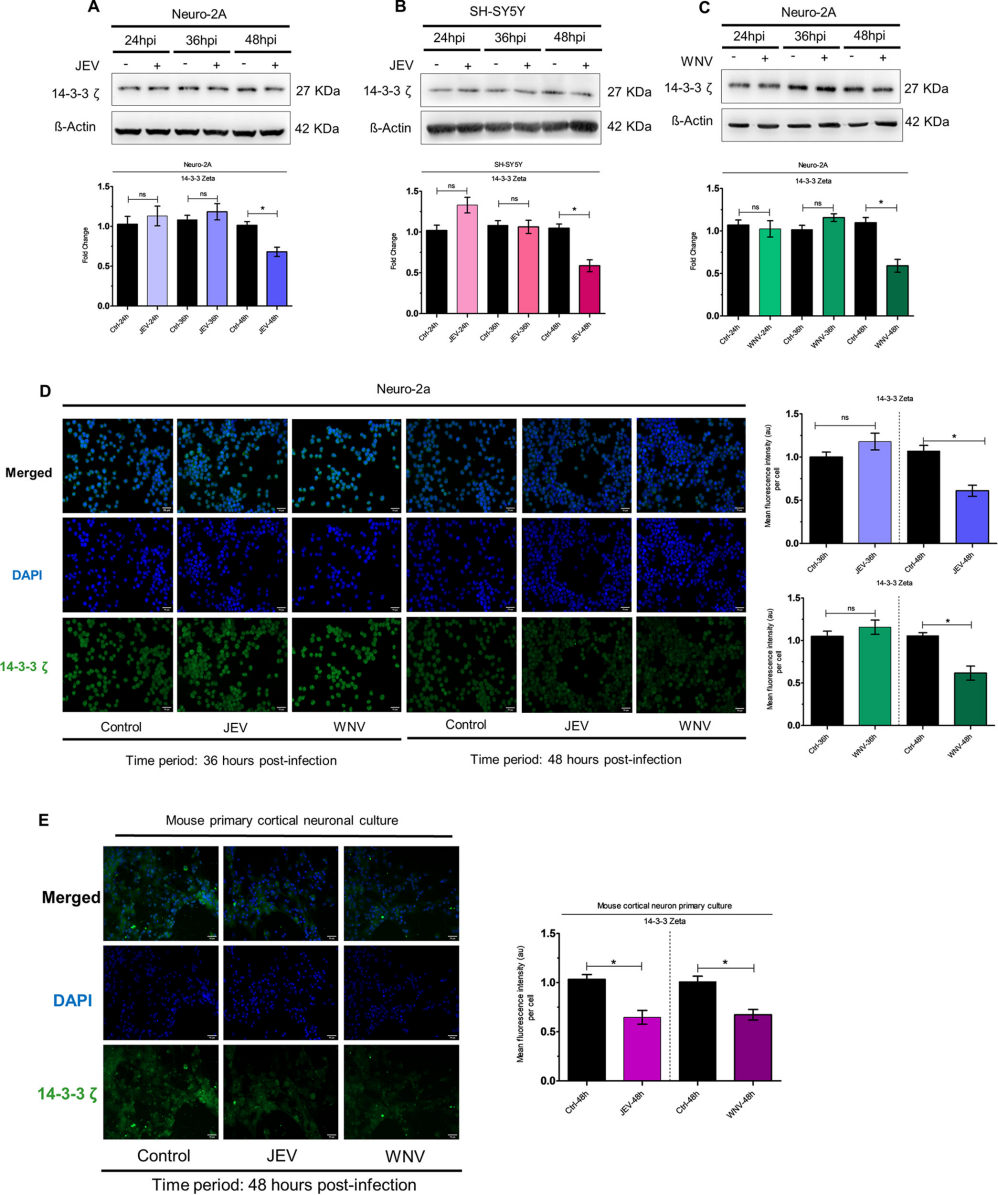
JEV/WNV infection of neuronal cells reduce 14-3-3ζ protein abundance. Time-dependent expression analysis of 14-3-3ζ protein was performed using protein isolated from JEV-infected (MOI 3) Neuro-2A cells (A) or SH-SY5Y cells (B) and WNV-infected (MOI 3) Neuro-2A cells (C) for indicated time periods. ß-actin was used as a loading-control. Densitometric analysis of immunoblots demonstrating time-dependent kinetics of 14-3-3ζ (normalized to ß-actin) in JEV-infected Neuro-2A (A, bottom: ratio of 14-3-3ζ to ß-actin), JEV-infected SH-SY5Y (B, bottom: ratio of 14-3-3ζ to ß-actin) and WNV-infected Neuro-2A (C, bottom: ratio of 14-3-3ζ to ß-actin). Molecular weight in kDa of analyzed molecules has been presented beside the respective immunoblots. (D) Immunocytochemical study using Neuro-2A-cells infected with JEV/WNV (MOI 3) for 36 and 48 h demonstrates 14-3-3ζ abundance. Scale bar shown in micrographs measures 50 μm (×20 magnification). Mouse primary cortical neuronal cells were subjected to immunofluorescence microscopy in an effort to evaluate 14-3-3ζ expression level upon infection by JEV/WNV (MOI 3) for 48 h. Scale bar indicated in micrographs measures 50 μm (×20 magnification). Quantitative analysis of micrographs demonstrating effect of Neuro-2A infection by JEV (D, right top: blue bar), WNV (D, right bottom: green bar) and mouse cortical neuron primary culture infection by JEV/WNV (E, right) for respective time points as denoted in the figure on 14-3-3ζ expression. Bar graphs denote data in the form of mean ± SD collected from three independent experiments with similar results.*, *P* < 0.05, ns=non-significant, by two-tailed Student's *t* test.

Immunoblot studies demonstrating time-dependent 14-3-3ζ expression kinetics upon infection by JEV (Fig. 6A) and WNV (Fig. 6C) in Neuro-2A cells, and JEV (Fig. 6B) and WNV (Fig. S5H) in SH-SY5Y cells showed downregulation at 48 hpi. Similar findings were also revealed upon immunocytochemical staining of Neuro-2A cells for 14-3-3ζ protein upon JEV/WNV infection (Fig. 6D) whereupon virus infection resulted in reduction of 14-3-3ζ expression at 48 hpi. Staining of mouse primary cortical neuronal cells for 14-3-3ζ exhibited JEV/WNV-induced downregulation at 48 hpi (Fig. 6E), underscoring the possibility that JEV/WNV-induced miR-451a might play role in modulation of 14-3-3ζ protein levels in neuronal cells.

### JEV/WNV-induced mir-451a upregulation modulates 14-3-3ζ abundance in neuronal cells.

**FIG 7:**
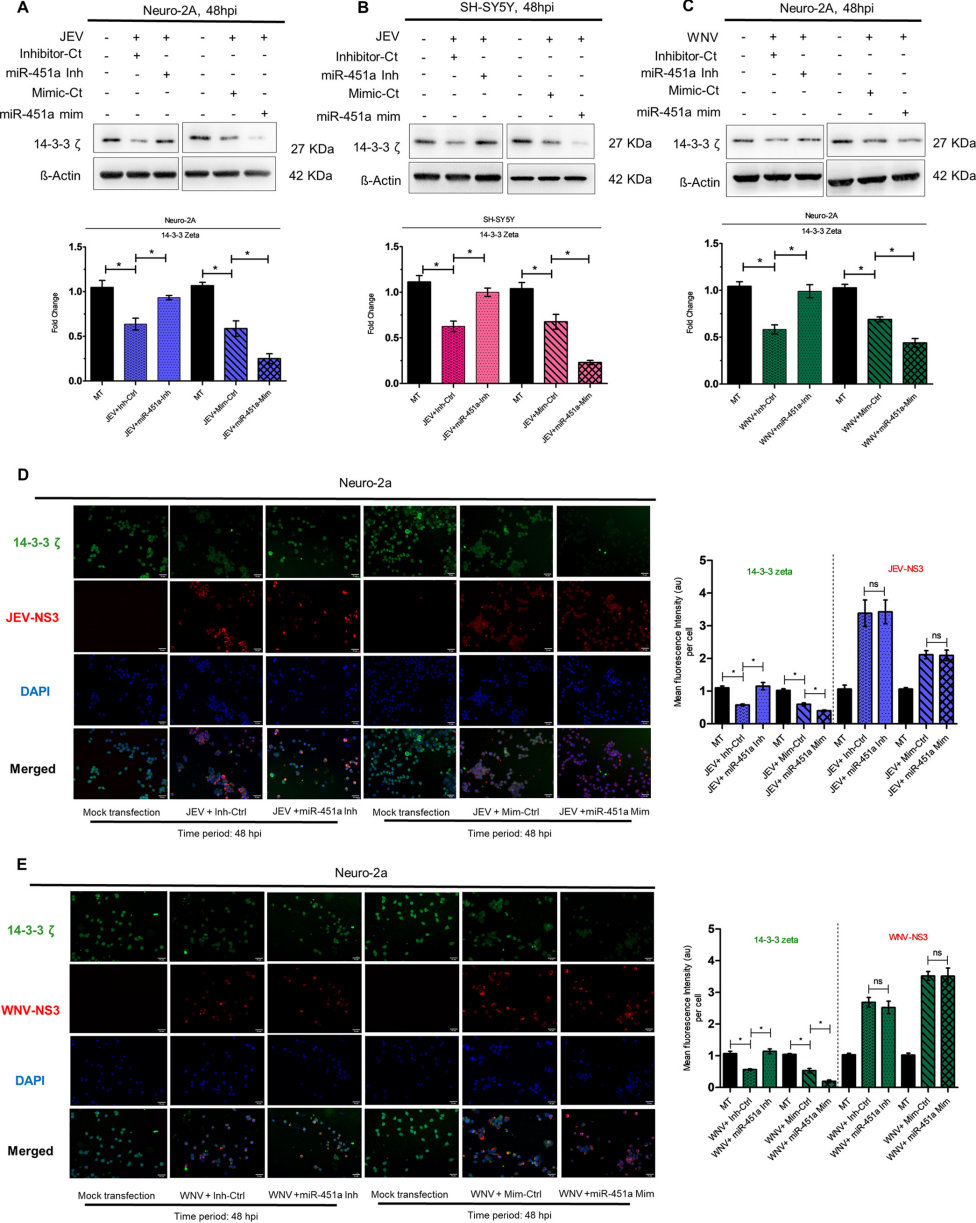
miR-451a modulates 14-3-3ζ abundance in neurons upon infection by JEV/WNV. Immunoblotting analysis of 14-3-3ζ protein expression in JEV-infected (MOI 3) Neuro-2A cells(A) and SH-SY5Y cells (B), and WNV-infected (MOI 3) Neuro-2A cells (C) for 48 h following transfection with miR-451a inhibitor/inhibitor-control and miR-451a mimic/mimic-control as denoted in the figure. Represented blots are data from three experimental replicates with similar data. ß-actin served as a loading control. Densitometric analysis of immunoblots denoting effects of miR-451-a inhibitor/inhibitor-control/miR-451a mimic/mimic-control transfection in JEV-infected (48 h) Neuro-2A (A, bottom: ratio of 14-3-3ζ to ß-actin), JEV-infected (48 h) SH-SY5Y (B, bottom: ratio of 14-3-3ζ to ß-actin), and WNV-infected (48 h) Neuro-2A cells (C, bottom: ratio of 14-3-3ζ to ß-actin) upon 14-3-3ζ expression (normalized to ß-actin). 14-3-3ζ expression and viral NS3 protein abundance evaluated by immunocytochemical analysis of Neuro-2A cells transfected with miR-451a inhibitor/inhibitor-control or miR-451a mimic/mimic-control for 24 h prior to being infected by JEV (D) or WNV (E) (MOI 3) for 48 h. The scale bar in each micrograph measures 50 μm; original (*×*20 magnification). Quantitative analysis of micrographs denote the effect of miR-451a inhibitor/inhibitor-control transfection upon 14-3-3ζ and viral protein NS3 expression exhibited by JEV-infected (D, right) and WNV-infected (E, right) Neuro-2A cells. Bar graphs denote data in the form of mean ± SD collected from three independent experiments with similar outcomes. *, *P* < 0.05, ns=non-significant, by two-tailed Student's *t* test.

Although JEV/WNV-infection leads to reduction of 14-3-3ζ protein in neuronal cells, it merely offers correlation rather than confirmatory evidence in favor of the role played by miR-451a upregulation in reducing 14-3-3ζ. Thus, we performed loss/gain-of-function experiments by transfecting JEV/WNV-infected Neuro-2A and SH-SY5Y cells with miR-451a inhibitor/mimic and their respective negative controls. Immunoblotting analysis using proteins isolated from JEV-infected (Fig. 7A) and WNV-infected (Fig. 7C) Neuro-2A cells, and JEV-infected (Fig. 7B) and WNV-infected SH-SY5Y cells (Fig. S5I) transfected with miR-451a inhibitor exhibited restoration of virus-mediated reduction of 14-3-3ζ abundance when compared to infected-cells transfected with miR-inhibitor-control. In congruence with findings stated above, overexpression of miR-451a in JEV/WNV-infected Neuro-2A and JEV-/WNV-infected SH-SY5Y cells using miR-451a mimic further decreased 14-3-3ζ levels in comparison to transfection by miR-mimic-control (Fig. 7A–7C and S5I). Immunofluorescence microscopic analysis of JEV-infected (Fig. 7D) and WNV-infected (Fig. 7E) Neuro-2A cells transfected with miR-451a inhibitor or mimic showed reversal or further strengthening of virus-induced 14-3-3ζ reduction in comparison to infected-cells transfected with respective negative-controls, whereas no alterations in viral-NS3 abundance upon miR-451a modulation by inhibitor/mimic, thus negating out any possibility for differential infection efficiency resulting in altered 14-3-3 ζ abundance. Hence, these findings provide strong evidence that in the context neuronal infection by JEV and WNV, miR-451a upregulation directly targets 14-3-3ζ transcript, decreasing its abundance at protein level.

### miR-451a-induced decrease in 14-3-3ζ abundance results in changes in phosphorylation of JNK, modulating neuronal apoptosis.

**FIG 8:**
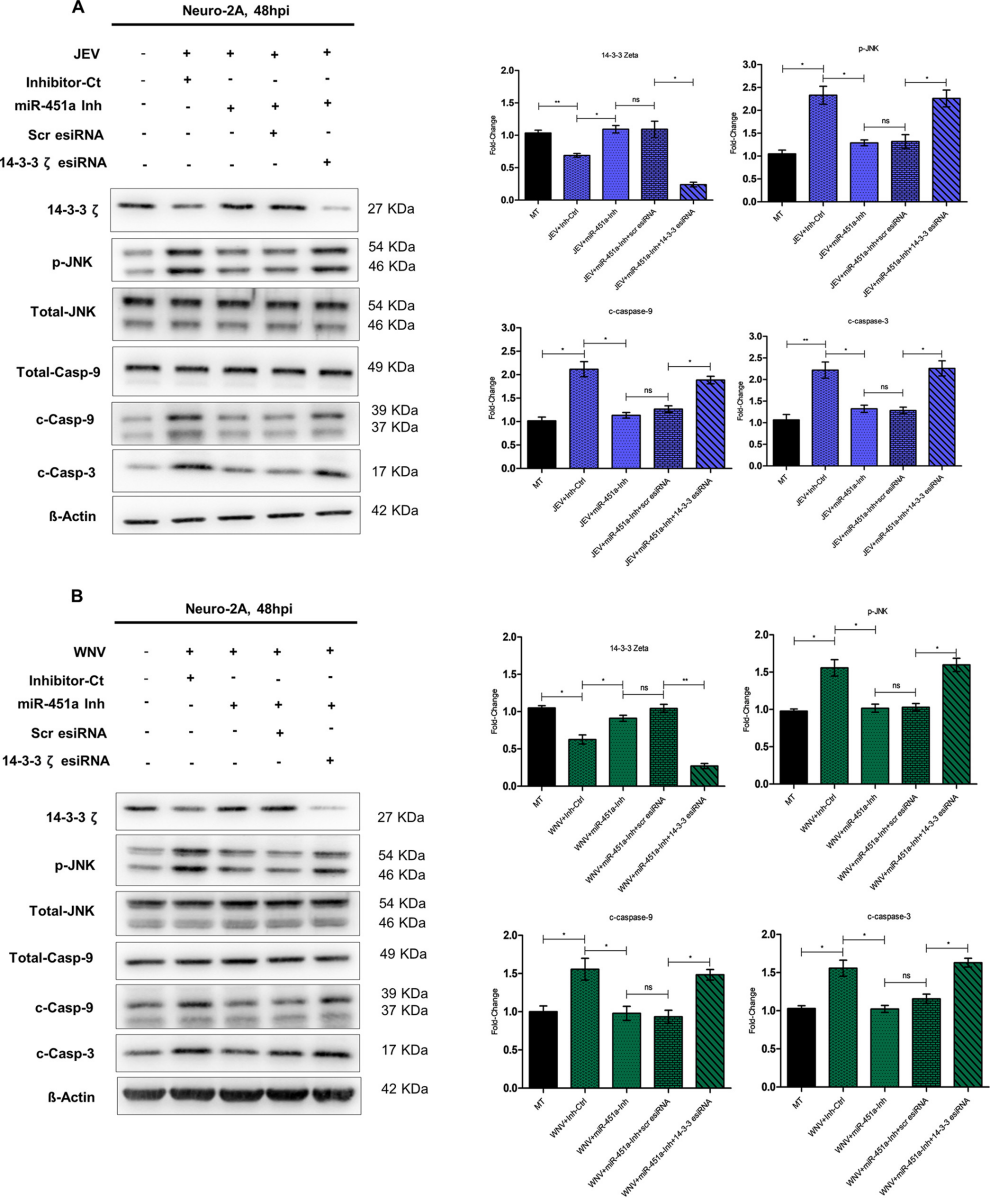
miR-451a-regulated 14-3-3ζ influences JNK phosphorylation upon infection by JEV/WNV, promoting neuronal apoptosis. Abundance of 14-3-3ζ, p-JNK, total-JNK, total-caspase-9, cleaved-caspase-9, and cleaved-caspase-3 was assessed upon immunoblotting analysis utilizing protein harvested from Neuro-2A-cells infected by JEV (A) or WNV (B) at MOI 3 for 48 h upon transfection with miR-451a inhibitor/inhibitor-control along with 14-3-3ζ-specific esiRNA/Scrambled-(Scr) esiRNA. The immunoblots represent data from three independent experiments with similar outcomes. ß-actin was used as a loading-control. Molecular weight of each probed molecules is stated in kDa beside the corresponding immunoblots. Densitometric analysis of immunoblots indicating effect of 14-3-3ζ esiRNA transfection in the context of miR-451a inhibition and JEV (A, right: ratio of 14-3-3ζ to ß-actin, ratio of phospho-JNK to total-JNK, ratio of cleaved-caspase-9 to total-caspase-9, and ratio of cleaved-caspase-3 to ß-actin) or WNV (B, right: ratio of 14-3-3ζ to ß-actin, ratio of phospho-JNK to total-JNK, ratio of cleaved-caspase-9 to total-caspase-9, and ratio of cleaved-caspase-3 to ß-actin) infection of Neuro-2A cells upon p-JNK (normalized with respect to total-JNK), cleaved-caspase-9 (normalized with respect to total-caspase-9),14-3-3ζ, cleaved-caspase-3 (normalized with respect to ß-actin). Data presented in bar graphs in the form of mean ± SD have been collected from three independent experiments with similar results. **, *P* < 0.001; *, *P* < 0.05; ns=non-significant by two-tailed Student's *t* test.

In order to evaluate whether reduction in 14-3-3ζ abundance owing to JEV/WNV-mediated miR-451a upregulation is indeed responsible for enhancement in JNK phosphorylation, resulting in neuronal apoptosis, we transfected JEV-infected (Fig. 8A) and WNV-infected (Fig. 8B) Neuro-2A cells with 14-3-3ζ esiRNA/enhanced green fluorescent protein (EGFP)-specific-esiRNA (Scr-esiRNA) along with miR-451a-inhibitor and assessed abundance of 14-3-3ζ, p-JNK, cleaved-caspase-9, total-caspase-9, and cleaved-caspase-3. A decrease in 14-3-3ζ expression associated with JEV/WNV infection was restored when miR-451a function was interfered with by employing miR-451a inhibitor. Transfection with 14-3-3ζ-esiRNA in the face of miR-451a inhibition and virus infection led to reduction in 14-3-3ζ. On the other hand, transfection of miR-451a-inhibitor-transfected and virus-infected Neuro-2a cells with EGFP-specific esiRNA did not result in decreased 14-3-3ζ protein abundance, thus referring to the specificity of 14-3-3ζ-esiRNA action. Reversal of miR-451a-inhibitor-induced 14-3-3ζ rescue in the context of JEV/WNV infection by 14-3-3ζ-esiRNA transfection was found to be accompanied by reversal of JNK phosphorylation inhibition following miR-451a inhibition, pointing toward the role of 14-3-3ζ as a mediator aiding in modulating p-JNK abundance due to JEV/WNV-mediated miR-451a upregulation. Concomitant with restoration of JNK-phosphorylation inhibition, 14-3-3ζ-esiRNA transfection of JEV/WNV-infected and miR-451a-inhibitor-transfected cells led to an increase in abundance of apoptotic molecular markers like cleaved-caspase-3 and -9 in comparison to JEV/WNV-infected cells transfected with miR-451a-inhibitor as well as negative-control esiRNA. Thus, the experiments involving manipulation of 14-3-3ζ abundance in the direction opposite to that conferred by miR-451a inhibition in the context of JEV/WNV infection conclusively proves the identity of 14-3-3ζ as an integral part of JEV/WNV infection-induced molecular circuitry, which also involves miR-451a and p-JNK and eventually paves way for neuronal apoptosis.

## DISCUSSION

Viral infection-induced cellular death by activation of apoptotic machinery has been considered to be a host-defense mechanism aimed at curtailing viral propagation. However, tissue like central nervous system (CNS) owing to poor regenerative capacity upon neuronal loss in the context of any microbial invasion does not fare well from the perspective of disease prognosis of patients. Besides acute neurological complications displayed by JEV/WNV-infected patients, individuals, upon recovery, exhibit a multitude of symptoms ranging from motor deficits in both upper and lower limbs, as well as cognitive and language impairment (30, 31), which can be attributed to brain-region-specific neuron loss. Although treatments are subjected to alleviate symptom exaggeration, to date, no specific intervention exists to limit virus-mediated CNS damage.

Evidence in favor of miRNAs modulating JEV-induced neuroinflammation (32) and WNV-induced cell death (33) point toward the potential for these miRNAs to be used as therapeutic targets in an attempt to combat arthropod-borne neurotropic virus infection. Apoptosis is a process by which cells sense persistent threat and its associated damage, eventually activating a plethora of effector molecules leading to an ordered cellular demise. Albeit current discoveries have been successful in providing detailed insights into mechanistic pathways for apoptosis, owing to the robust apoptotic signaling network activated in the context of virus infection, attempts to swing the molecular balance of the network by blocking/inhibiting a single bcl-2 family member might be met with limited success. Due to these limitations, the need for deciphering molecular events upstream to initiation of apoptotic signaling following viral infection and the possibility for its use as a plausible therapeutic candidate urged us to investigate role of miRNA in flavivirus-induced neuronal apoptosis.

In our study, we studied the role of miR-451a upon JEV/WNV-mediated neuronal death. miR-451a was observed to be upregulated at late-stage flavivirus infection in neuronal cells, brain tissue of virus-infected BALB/c mouse-model, and human autopsy brain-tissue. Although miR-451a upregulation in JEV/WNV-infected murine and JEV-infected human brain tissue indicates the role of miR-451a in host-response, the effect of miR-451a upon neuronal apoptosis *in vivo* must be validated using a murine transgenic *in vivo* infection model offering provision for neuron-specific manipulation of miR-451a abundance in the face of JEV/WNV infection and studying its effect upon neuronal fate. Experimental evidence provided in this study show overexpression of miR-451a in neuronal cells promotes apoptosis upon JEV/WNV infection. The effect of miRNA-451a action upon neuronal apoptosis has been shown to be accompanied by lack of any changes in magnitude of virus propagation thus pointing toward the role for miR-451a in direct modulation of host signaling pathways rather than impacting viral infectious life cycle. ER-stress resulting from viral infection helps cells to keep pace with increased protein folding requirements. However, upon failure to meet the demand for enhanced protein folding capacity, cells undergo apoptosis. JEV-induced activation of PERK-CHOP axis has been demonstrated to play a crucial role in JEV-induced neuronal apoptosis(34). In addition to CHOP-induced cell death, JEV infection can activate IRE1/JNK pathway of ER stress response, which has also been shown to promote apoptosis (35). In an attempt to decipher whether miR-451a overexpression upon JEV infection is responsible for modulating ER stress response in favor for neuronal apoptosis, we studied the expression kinetics of multiple signaling molecules involved in JEV-induced ER stress pathways. Out of the multiple molecules studied (i.e., p-IRE-1α, p-PERK, ATF-4, CHOP and P-JNK), CHOP expression and JNK phosphorylation were observed to be upregulated at 48 hpi by JEV, which also coincided with miR-451a overexpression. Perturbation in JEV-induced miR-451a overexpression altered infection-associated JNK phosphorylation status but not CHOP abundance thus pointing toward role of miR-451a in modulating JNK signaling pathway. Similar causal relationship was observed between miR-451a expression and JNK phosphorylation upon WNV infection of neurons as well. Data indicating role of 14-3-3ζ protein in modulating JNK phosphorylation level and dual luciferase assay experiments demonstrating the potential of 14-3-3ζ 3′-UTR to act as target for miR-451a as primarily suggested by TargetScan website analysis in conjunction with causal link between miR-451a-upregulation and JNK phosphorylation urged us to evaluate the expression profile of 14-3-3ζ in neurons upon infection by JEV/WNV. The 14-3-3ζ protein was observed to be downregulated upon neuronal infection by JEV/WNV in a miR-451a-dependent fashion. The 14-3-3ζ protein belongs to 14-3-3 family consisting of 7 isoforms in mammals. The 14-3-3 group of proteins has been considered a signaling hub as evidenced by its role in regulating multiple cellular responses like oxidative stress, DNA damage, apoptosis (36). The 14-3-3 proteins by virtue of their interaction with multiple protein substrates have been demonstrated to control their sub-cellular localizations. 14-3-3 proteins upon formation of homo- or hetero-dimers interact with a diverse set of protein substrates, either restricting their sub-cellular localization or modulating their activity via change in protein-substrates’ conformation. Post-translational modifications or depletion of 14-3-3 proteins release their respective substrates, promoting a shift in their sub-cellular locations. 14-3-3 proteins being capable of binding to Bcl2-associated agonist of cell death (BAD) (37) and Bcl2-assocated X protein (BAX) (38), upon its own depletion, results in cytosol-to-mitochondrial shuttling of BAD and BAX, culminating into activation of intrinsic-apoptotic pathway. Experimental evidence provided in this study point toward the role of 14-3-3ζ protein in JEV/WNV-infected neurons in modulating p-JNK abundance along with caspase-9 and -3 activity. Upon depletion, 14-3-3ζ has been documented to release ASK1 protein from inhibition eventually promoting JNK phosphorylation (29). JNK activation upon phosphorylation can activate AP-1 transcription factor, leading to transcription of numerous proapoptotic factors (39). JNK activity can lead to enhanced stability and activity of p53 (40) and p73 (41), resulting in enhancement of p53- and p73-induced apoptotic activity. In addition to activating transcription of pro-apoptotic factors, activated JNK has been reported to modulate mitochondrial physiology by promoting cytochrome-c release to cytosol (42). Evidence shows activated-JNK is capable of activating BH3-interacting domain death agonist (BID) by cleaving the latter. Cleaved fragment of BID translocates to mitochondria and aid BAX in initiating apoptotic pathway activation. Phosphorylated-JNK has also been reported to activate apoptotic signaling by phosphorylation of BAD at position serine-128 (43). Thus, JNK upon being activated by phosphorylation can set off activation of this complicated network of apoptotic signaling, leading to cellular demise.

In conclusion, our findings demonstrate that miR-451a upregulation in JEV/WNV-infected neurons results in reduction of 14-3-3ζ abundance. Downregulation of 14-3-3ζ modulates JNK activation resulting in activation of intrinsic apoptotic pathway and neuronal death. In the light of acute neuronal damage elicited by JEV/WNV infection followed by neurological sequelae experienced by recovered patients, our study provides us with a possible novel therapeutic opportunity in reducing disease burden. However, since this study has employed a reductionist model comprising of neuronal cell-culture, animal studies with provision for neuron-specific miR-451a/14-3-3ζ inhibition or overexpression in the face of JEV/WNV infection and evaluating its effect upon neuronal physiology are warranted prior to its application in clinical settings.

## MATERIALS AND METHODS

### Mice.

Both male and female BALB/c mice (purchased from Jackson Laboratory, Bar Harbor, ME) were harbored together with their respective mothers under a 12-h light/12-h dark cycle maintained at constant temperature and humidity in the animal facility of NBRC. Animals were handled with utmost care following recommended animal-handling practice guidelines issued by the Committee for the Purpose of Control and Supervision of Experiments on Animals, Ministry of Environment and Forestry, Government of India. All the experiments were performed following approval from the Animal Ethics Committee of National Brain Research Centre (NBRC/IAEC/2017/30).

### JEV and WNV infection in mice.

10-day-old BALB/c mice employed for the purpose of *in vivo* experiments were divided into two groups, mock-infected (intraperitoneal administration of equal volume of 1X-PBS solution) and virus-infected. Mice were infected with JEV/WNV (3 × 10^5^ virus particles) via intraperitoneal route. 5 days following virus administration and upon appearance of symptoms, BALB/c-mice were euthanized and brain samples were collected for further analysis.

### miRNA isolation from human autopsy brain-tissue section.

Brain tissue-sections were collected from age-matched deceased individuals as a result of motor-vehicle accidents with least-possible trauma to brain. Autopsy brain tissue sections were procured from Human Brain Bank, National Institute of Mental Health and Neuroscience (NIMHANS), Bangalore, following institutional guidelines. RNA was isolated from paraffin-embedded autopsy brain tissue-sections of JEV-infected individuals. Briefly stated, deparaffinization was performed, as per recommendations provided by published protocol (44), by immersing tissue sections in mineral oil at 95°C for 2 min until paraffin was removed. Tissue sections were then washed thoroughly using RNAlater solution (Sigma-Aldrich, USA). Tissue sections were then homogenized using Qiazol (Qiagen, USA), followed by RNA isolation using miRNeasy kit (Qiagen, USA).

### Primary cortical-neuron culture.

2-day old BALB/c mice were decapitated, followed by collection of brain cortical tissue, aided by dissection microscope. Following collection of brain cortical tissue, they were subjected to digestion by trypsin- and DNase-treatment thus preparing a single-cell suspension. The solution was passed through nylon mesh possessing 127 μm-sized pores, followed by centrifugation at 500 × *g* for 2 min. Pellet was collected, and cell number was counted using trypan blue stain and hemocytometer. Equal number of cells were plated in poly-d-Lysine-coated plates/slides followed by maintenance using neurobasal media supplemented with 2 nM l-glutamine, fetal bovine serum, horse serum, and Penicillin-streptomycin. Following 2 days of maintenance of cells in the aforementioned media, half of the media was replaced by neurobasal media along with N2-, B27-supplement, penicillin-streptomycin. Arabinoside-C (Ara-C) (Sigma-Aldrich, USA) was added to the media such that its final concentration achieved was 20 μM for elimination of glial cells. Following the entire procedure, the cells were maintained at 37°C and 5% CO_2_.

### Virus propagation and titration.

GP78 and Eg101 strains (acquired from National Institute of Virology, Pune, India) of JEV and WNV, respectively, were propagated using 2-day old BALB/c mice. Following appearance of symptoms (5–6 days followed by infection by both JEV and WNV), brain tissues were collected and viral suspension was prepared. Virus suspension was stored at –80°C for future use. JEV and WNV titer was estimated using porcine stable (PS) kidney cells and kidney epithelial cells from green monkey (veroE6 cells), respectively, by employing plaque assay. Serial dilutions of purified JEV/WNV-containing solutions were used to infect monolayer of PS & VeroE6 cells respectively. Following infection for 2 h, cells were washed with sterile 1X phosphate-buffered saline (PBS) and were covered with solution containing 1% low melting-agarose, 1X MEM and fetal bovine serum followed by its solidification. Infected-cell monolayer covered with agarose overlay was incubated for 96 h at 37°C in the presence of 5% CO_2_. Overlay was then removed, and cells were stained using crystal violet for detection of plaques.

### Cell-culture.

Mouse neuroblastoma cell-line Neuro-2A (kind gift from Dr. Nihar Ranjan Jana, IIT-Kharagpur) was maintained using Dulbecco modified Eagle medium (DMEM) supplemented with 10% fetal bovine serum, penicillin (100U/mL) and streptomycin (100 μg/mL). Human neuroblastoma cell-line SH-SY5Y (kind gift from Dr. S. Levison, Rutgers University, New Jersey Medical School, NJ) was maintained using DMEM/F12 media supplemented with 10% fetal bovine serum, penicillin (100U/mL) and streptomycin (100 μg/mL). Both the cells were incubated at 37°C and 5% CO_2_.

Mouse neuroblastoma cell line Neuro-2A was authenticated in November 2018 by Applied Biosystems. Human neuroblastoma cell line SH-SY5Y was authenticated in January 2019 by DNA Forensics Laboratory Pvt. Ltd., New Delhi, India.

### Infection of cells by viruses.

All the cells were seeded at density as per requirements of the experiments and, upon reaching 75% confluence, were treated with serum-free media for 2 h prior to addition of virus. JEV GP78 or WNV Eg101 strains were then used at 3 multiplicity of infection (MOI) to infect cells for another 2 h. Following that, cells were washed twice with 1X PBS-solution to remove the loosely adsorbed virus particles. Cells were then maintained using maintenance media supplemented with fetal bovine serum for diverse time points as indicated in different experiments. Throughout the entire process, cells were incubated at 37°C and 5% CO_2_.

### Apoptotic-miRNA array.

RNA was isolated from JEV-infected Neuro-2A cells using miRNeasy kit (Qiagen, CA, USA) followed by cDNA preparation utilizing MiScript II RT kit (Qiagen, CA, USA). cDNA was then subjected to MiScript miRNA-PCR array (Qiagen, product number: 331221, catalogue number: MIMM-114ZA) using miScript SYBR Green PCR Kit (Qiagen, CA, USA). miRNA array data were analyzed employing Qiagen data analysis software available online.

### Transfection of cells using miRNA-inhibitor and miRNA-mimic.

In order to inhibit or overexpress mature-miR-451a, we transfected cells with miR-451a inhibitor (small RNA molecule targeting mouse as well as human endogenous mature miR-451a, Qiagen, CA, USA, catalogue number: YI04101735-ADA) and miR-451a mimic (small RNA molecules mimicking mature mouse and human endogenous miR-451a, Qiagen, CA, USA, catalogue number: YM00471387-ADA), respectively. miRNA-inhibitor-control/-mimic-control were similarly transfected using Opti-MEM-media (Gibco, USA) and transfection reagent Lipofectamine-2000 (Invitrogen, USA) as per manufacturer’s protocol as matched negative controls. An equal volume of Opti-MEM and Lipofectamine-2000 without any nucleic acid were used for mock-transfected cells.

### Plasmid construction.

A 1001-base-pair long fragment belonging to mouse 14-3-3ζ-3′-UTR possessing miR-451a-binding site was amplified using primers; forward: 5′- CTCGAGCTAGGATCGTGTGTGTCTGCG-3′, reverse: 5′-GTCGACGTGAGCCTGTGCCCAACATGG-3′, followed by its cloning into XhoI and SalI sites downstream of firefly-luciferase gene of pmirGlo reporter plasmid (Promega). Site-directed mutagenesis in the miR-451a-binding site of 14-3-3ζ 3′-UTR was performed using primers; forward: 5′-AGGGCAGACCTTTGGCACATTCCATTATTTGT-3′, reverse: 5′-GGAATGTGCCAAAGGTCTGCCCTTACCAGAGT-3′, utilizing a PCR-based method employing Phusion High-Fidelity DNA polymerase (Thermo Fisher). Subsequently, all the constructs were sequenced at Regional Centre for Biotechnology (RCB), India.

### Luciferase reporter assay.

Neuro-2A cells were transfected with pmirGlo reporter plasmid (Promega) possessing wild-type/mutated 14-3-3ζ 3′-UTR along with either miR-451a inhibitor/inhibitor-control or miR-451a mimic/mimic-control using Opti-MEM and Lipofectamine-2000. Following 24 h of transfection, cells were harvested and subjected to luciferase assay using luciferase assay system (Promega, catalogue number: E4030) according to manufacturer’s recommendation. Firefly-luciferase activity has been normalized using renilla-luciferase activity coded by the same pmirGlo reporter plasmid.

### Transfection of endoribonuclease-prepared small-interfering RNAs (esiRNAs) and plasmids.

esiRNA specific for mouse-14-3-3ζ (purchased from Sigma-Aldrich, USA, catalogue number: EMU000731-50UG) and pmirGlo plasmid were transfected into Neuro-2A employing transfection reagent Lipofectamine-2000 (Invitrogen, USA) and Opti-MEM (Gibco, USA). EGFP-specific esiRNA (Sigma-Aldrich, USA, catalogue number : EHUEGFP-50UG) was used to transfect neuro-2A cells as matched negative-control against 14-3-3ζ-specific esiRNA. Transfection efficiency of 14-3-3ζ-specific esiRNA was estimated utilizing immunoblotting for 14-3-3ζ.

### Quantitative PCR (qRT-PCR).

RNA was isolated using miRNeasy kit (Qiagen, USA) followed by preparation of cDNA by MiScript II RT kit (Qiagen USA) and PCR was carried on employing miScript SYBR green PCR Kit (Qiagen, USA). miRNA abundance was estimated using primer targeting both mouse and human mature-miR-451a, SNORD68 primer (Qiagen, USA) targeting both mouse and human SNORD68 (Qiagen, USA). Relative abundance of mature-miR-451a was elucidated using the delta-delta C_t_ method using SNORD68 abundance as a loading control.

### Western blotting.

Cell-derived protein lysate was prepared using lysis buffer containing 10 mM tris-HCl (pH 8.0), 1% Triton X-100, 150 mM NaCl, 0.5% NP-40, 1 mM EDTA, 0.2% EGTA, 0.2% sodium orthovanadate, and protease-inhibitor cocktail (Sigma-Aldrich, USA). Lysate was then subjected to centrifugation at 4°C at 12,000 × *g* for 30 min. Supernatant was collected and subjected to bicinchoninic acid (BCA) (Sigma-Aldrich, USA) assay for estimation of protein concentration. An identical amount of protein from each sample was heated at 95°C along with sample-buffer for 5 min followed by their separation utilizing SDS-PAGE. Proteins were then transferred to nitrocellulose membrane followed by blocking using non-fat milk-solution or bovine serum albumin, BSA (Sigma-Aldrich, USA) solution. Membrane containing translocated proteins were then probed using primary antibodies against 14-3-3ζ (1:5000, Santa Cruz) (initial aliquot gifted by Dr. Sagar Sengupta, National Institute of Immunology, New Delhi), ß-actin (1:10,000 Sigma-Aldrich), p-IRE1-α (1:1000, Novus), total-IRE1-α (1:1000, Novus), p-PERK (1:2000, Cell Signaling Technology), total-PERK (1:2000, Cell Signaling Technology), ATF-4 (1:2000, Cell Signaling Technology), CHOP (1:2000, Cell Signaling Technology), p-JNK (1:2000, Cell Signaling Technology), total-JNK (1:2000, Cell Signaling Technology), caspase-9 (1:2000, Cell Signaling Technology), cleaved-caspase-3 (1:1000, Cell Signaling Technology), JEV-NS3 (1:5000, Genetex), WNV-NS3 (1:2000, R&D Systems). Peroxidase-conjugated rabbit and mouse-specific secondary antibody (1:5000, Cell Signaling Technology) was used for chemiluminescent detection using Chemigenius bioimaging system (Uvitec Cambrige, Cleaver Scientific, United Kingdom).

### TUNEL staining.

TUNEL assay was carried out using DeadEnd fluorometric TUNEL system (Promega, USA) according to manufacturer’s instructions. Briefly, 2 × 10^5^ cells were seeded on 8-chamber slides. Following respective treatments, cells were fixed using paraformaldehyde and incubated with recombinant terminal deoxynucleotidyl transferase and nucleotide mix. Cells were then washed before mounting with DAPI-containing mounting media (Vector laboratories, USA) and were observed under Zeiss Apotome microscope (Carl Zeiss, Germany) and images were captured at ×10 magnification.

### Annexin V and propidium Iodide staining.

Cells undergoing treatment under multiple conditions were harvested using ice-cold 1X PBS solution and then resuspended using 1X annexin-binding buffer provided with FITC-Annexin V apoptosis detection kit (BD Biosciences, USA). Following manufacturer’s instructions, cells were incubated with FITC-conjugated annexin and propidium iodide solution and analyzed using BD FACSVerse (USA).

### Immunocytochemistry.

Neuro-2A or mouse primary cortical-neuronal cells were cultured on 4-chamber slides and infected with JEV/WNV as indicated in experiments. Following infection, cells were fixed using 4% paraformaldehyde solution. Blocking was performed using 10% BSA solution followed by incubation with FITC-conjugated primary antibody against 14-3-3ζ (Santa Cruz), JEV NS-3 (Gentex, USA) and WNV NS-3 (R&D Systems, UK), at 4°C for overnight. This was followed by washing using PBS-solution containing detergent Triton-X, then cells were incubated with anti-rabbit and anti-mouse antibodies tagged with Alexa Fluor 594 at room temperature for 1 h. Cells were then subjected to washing using PBS-solution containing detergent Triton-X (Sigma-Aldrich, USA) and mounted using DAPI-containing mounting media (Vector Laboratories, USA). Cells were observed under Zeiss Apotome microscope (Carl Zeiss, Germany) and images were captured at ×20 magnification.

### Statistical analysis.

Statistical analysis was performed using GraphPad Prism 5.0 software. Student's *t* test (two-tailed) was performed for evaluating statistical significance of difference between 2-groups. All the measurement data reported in the manuscript have been represented as mean ± SD.
