# Supplementary material for: miR-451a Regulates Neuronal Apoptosis by Modulating 14-3-3ζ-JNK Axis upon Flaviviral Infection
Source: mSphere. 2022 Jun 21;7(4):e00208-22. doi: 10.1128/msphere.00208-22 (PMC9429931; doi:10.1128/msphere.00208-22)
Supplement: TABLE S1 [file msphere.00208-22-s0001.pdf]

Supplementary Table: 1, Expression data of miRNA in neurons upon JEV infection.

**miRNA name**	**Relative fold Change**	***P-value***
mmu-let-7a-5p	0.96	0.753446
mmu-let-7c-5p	0.97	0.396516
mmu-let-7e-5p	1.02	0.506136
mmu-let-7g-5p	1.02	0.33142
mmu-miR-101a-3p	1.3	0.050812
mmu-miR-106b-5p	0.91	0.036762
mmu-miR-122-5p	0.98	0.577617
mmu-miR-125a-5p	1.04	0.31025
mmu-miR-125b-5p	0.92	0.045782
mmu-miR-128-3p	1.05	0.204907
mmu-miR-133a-3p	1	0.054275
mmu-miR-133b-3p	0.98	0.577617
mmu-miR-134-5p	1.19	0.290283
mmu-miR-141-3p	0.98	0.577617
mmu-miR-143-3p	0.9	0.104732
mmu-miR-144-3p	1.02	0.888292
mmu-miR-145a-5p	0.84	0.005482
mmu-miR-146a-5p	0.85	0.032354
mmu-miR-148a-3p	0.93	0.509268
mmu-miR-153-3p	0.85	0.000528

mmu-miR-155-5p	0.98	0.577617
mmu-miR-15a-5p	0.97	0.091484
mmu-miR-15b-5p	0.98	0.587355
mmu-miR-16-5p	0.98	0.463509
mmu-miR-17-5p	0.96	0.283228
mmu-miR-181a-5p	0.87	0.052159
mmu-miR-181b-5p	0.95	0.038985
mmu-miR-181c-5p	0.86	0.103826
mmu-miR-181d-5p	0.94	0.177111
mmu-miR-183-5p	1.57	0.078161
mmu-miR-185-5p	0.98	0.721462
mmu-miR-186-5p	0.97	0.330925
mmu-miR-192-5p	0.99	0.865071
mmu-miR-194-5p	0.97	0.255591
mmu-miR-195a-5p	0.98	0.676294
mmu-miR-1a-3p	1.07	0.663588
mmu-miR-200c-3p	0.6	0.082836
mmu-miR-203-3p	1.97	0.000341
mmu-miR-204-5p	1.14	0.419405
mmu-miR-205-5p	1.23	0.048839
mmu-miR-206-3p	0.98	0.577617
mmu-miR-20a-5p	0.95	0.269971
mmu-miR-21a-5p	0.94	0.241312

mmu-miR-210-3p	0.99	0.826484
mmu-miR-214-3p	0.9	0.005633
mmu-miR-218-5p	1	0.897238
mmu-miR-221-3p	1.02	0.781708
mmu-miR-222-3p	1.27	0.363371
mmu-miR-23a-3p	1.01	0.596705
mmu-miR-24-3p	1.01	0.786623
mmu-miR-25-3p	1.05	0.25202
mmu-miR-26a-5p	1	0.962727
mmu-miR-26b-5p	1.06	0.322705
mmu-miR-27a-3p	1.05	0.299538
mmu-miR298-5p	0.67	0.000111
mmu-miR-29a-3p	1.09	0.048376
mmu-miR-29b-3p	1.2	0.253167
mmu-miR-29c-3p	1.06	0.066093
mmu-miR-30a-5p	0.95	0.293773
mmu-miR-30b-5p	1	0.976067
mmu-miR-30c-5p	1.03	0.179838
mmu-miR-30d-5p	0.94	0.197234
mmu-miR-30e-5p	0.96	0.242047
mmu-miR-31-5p	1.02	0.776031
mmu-miR-32-5p	0.93	0.03757
mmu-miR-338-3p	1.26	0.466962
mmu-miR-34a-5p	1.01	0.872139

mmu-miR-34c-5p	0.98	0.577617
mmu-miR-351-5p	0.86	0.717421
mmu-miR-365-3p	0.9	0.11131
mmu-miR-378a-3p	0.91	0.060096
mmu-miR-409-3p	1.08	0.561543
mmu-miR-449a-5p	1.01	0.642826
mmu-miR-451a	2.94	0.011876
mmu-miR-4661-3p	1.15	0.068807
mmu-miR-491-5p	0.6	0.001293
mmu-miR-497a-5p	0.97	0.657375
mmu-miR-503-5p	0.87	0.123504
mmu-miR-542-3p	0.98	0.577617
mmu-miR-708-5p	1.44	0.030674
mmu-miR-7a-5p	1.16	0.001169
mmu-miR-9-5p	1.21	0.232862
mmu-miR-92a-3p	0.85	0.163258
mmu-miR-98-5p	1.04	0.544681
