# Supplementary figures and images for: miR-451a Regulates Neuronal Apoptosis by Modulating 14-3-3ζ-JNK Axis upon Flaviviral Infection
Source: mSphere. 2022 Jun 21;7(4):e00208-22. doi: 10.1128/msphere.00208-22 (PMC9429931; doi:10.1128/msphere.00208-22)

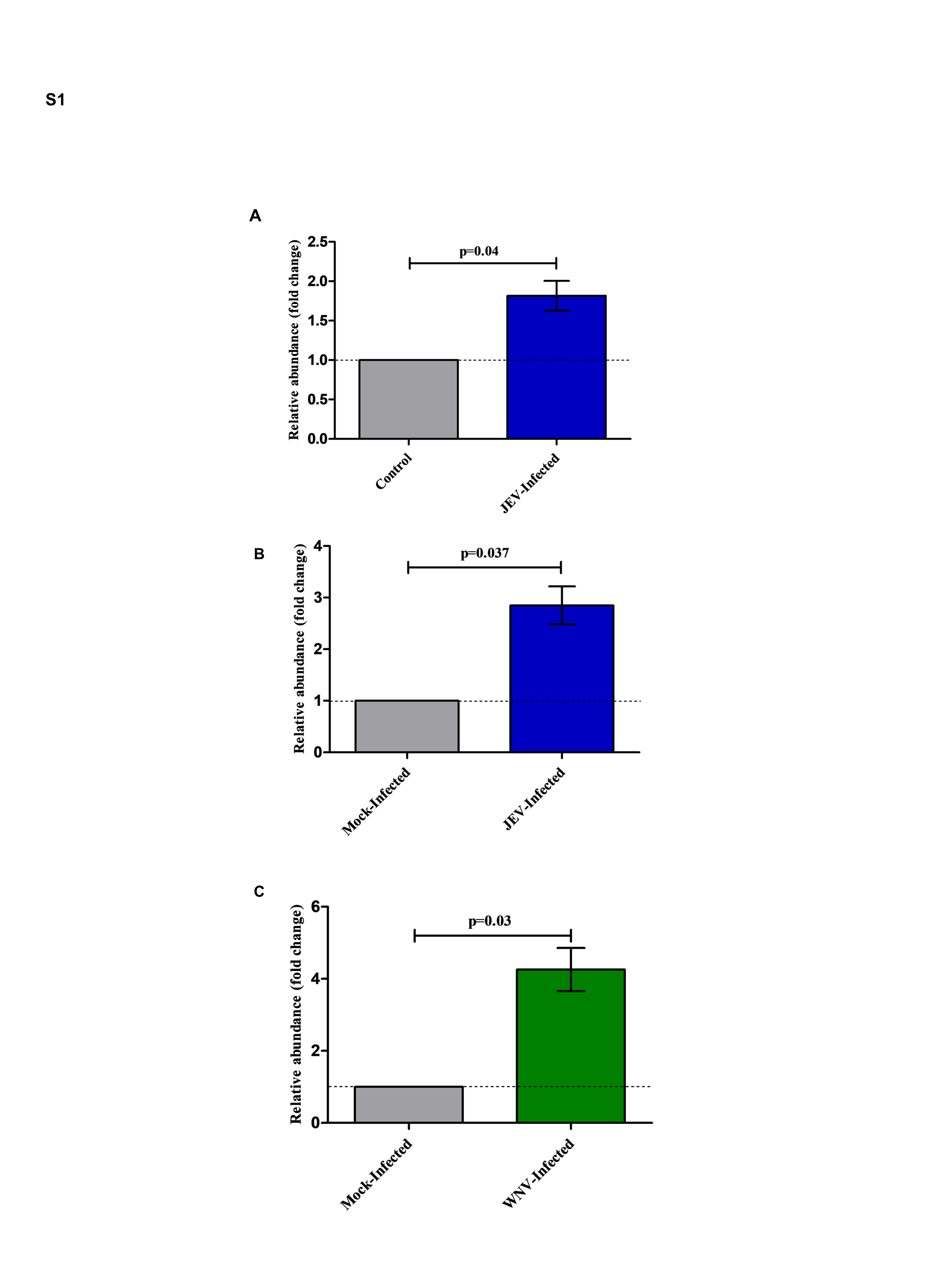

Supplement: FIG S1 [file msphere.00208-22-s0002.tif]

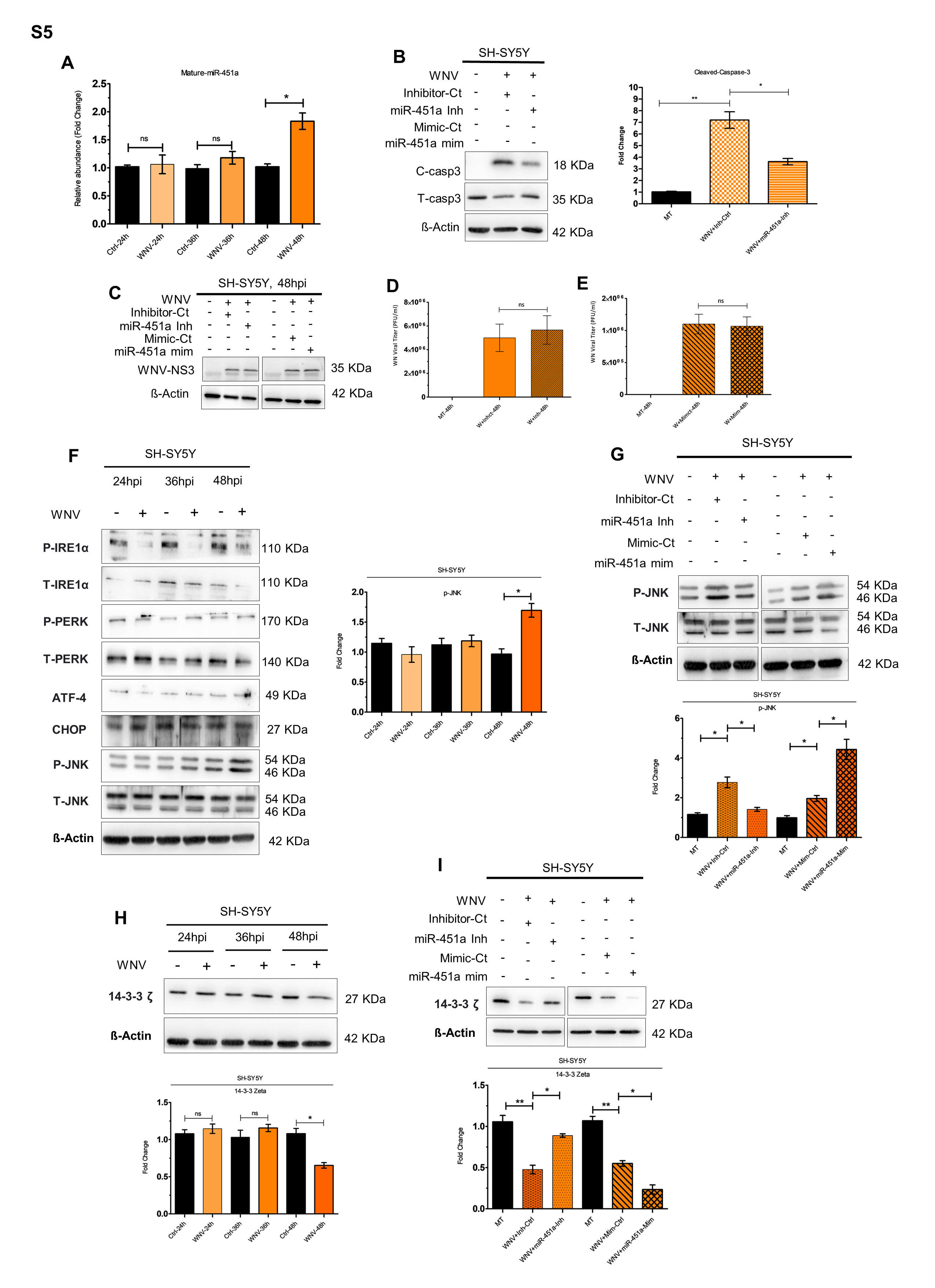

Supplement: FIG S5 [file msphere.00208-22-s0006.tif]

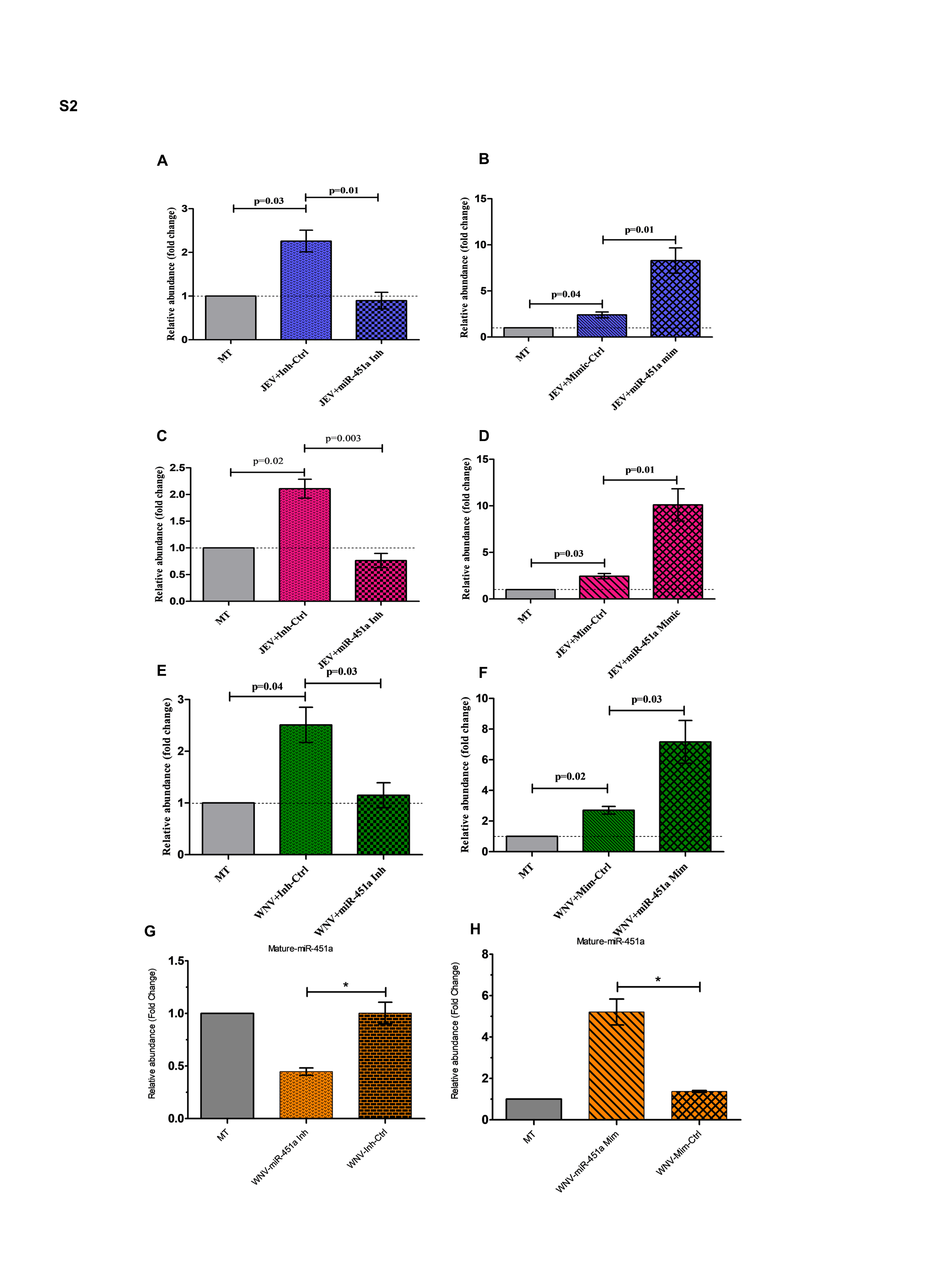

Supplement: FIG S2 [file msphere.00208-22-s0003.tif]

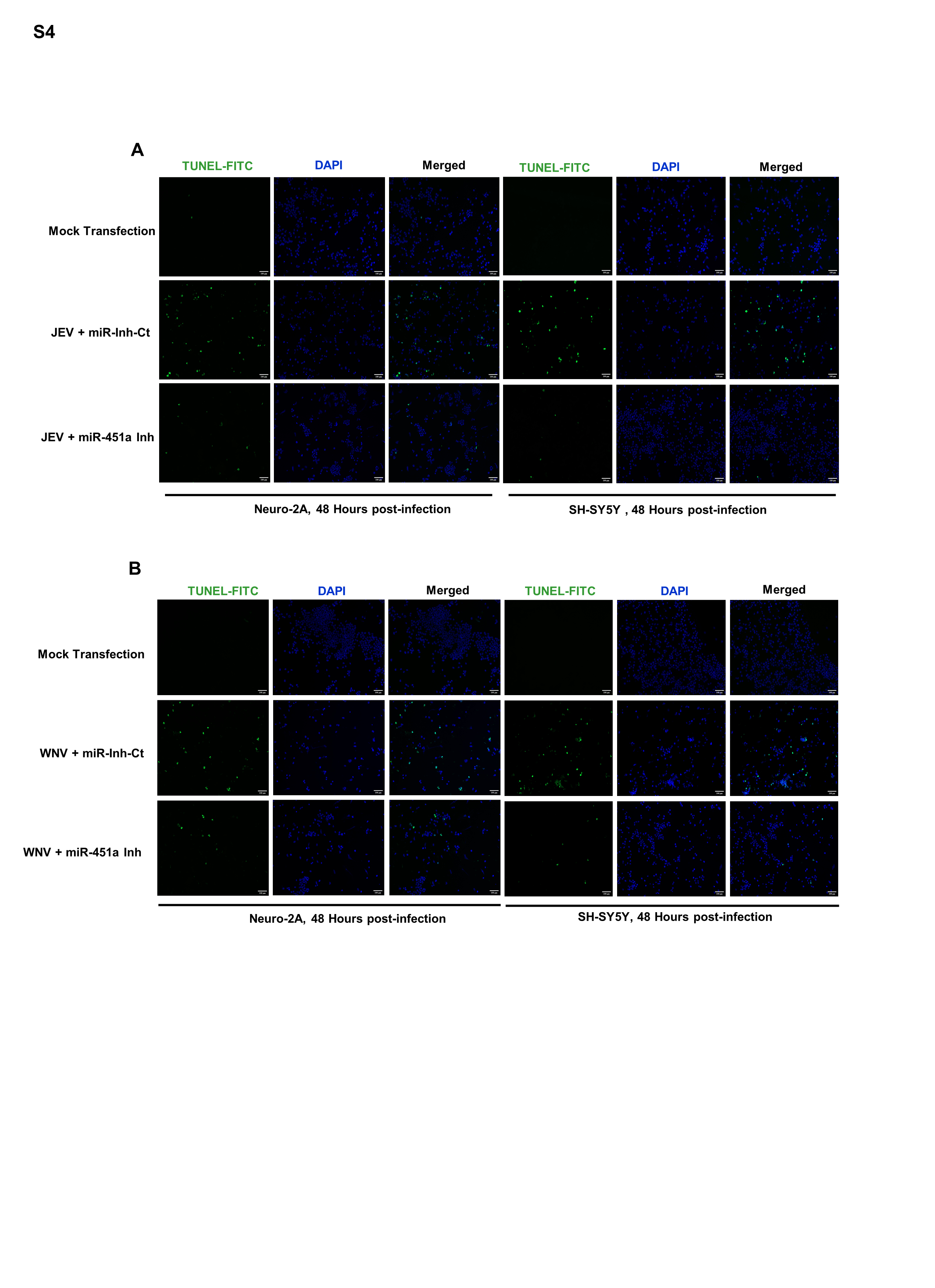

Supplement: FIG S4 [file msphere.00208-22-s0005.tif]

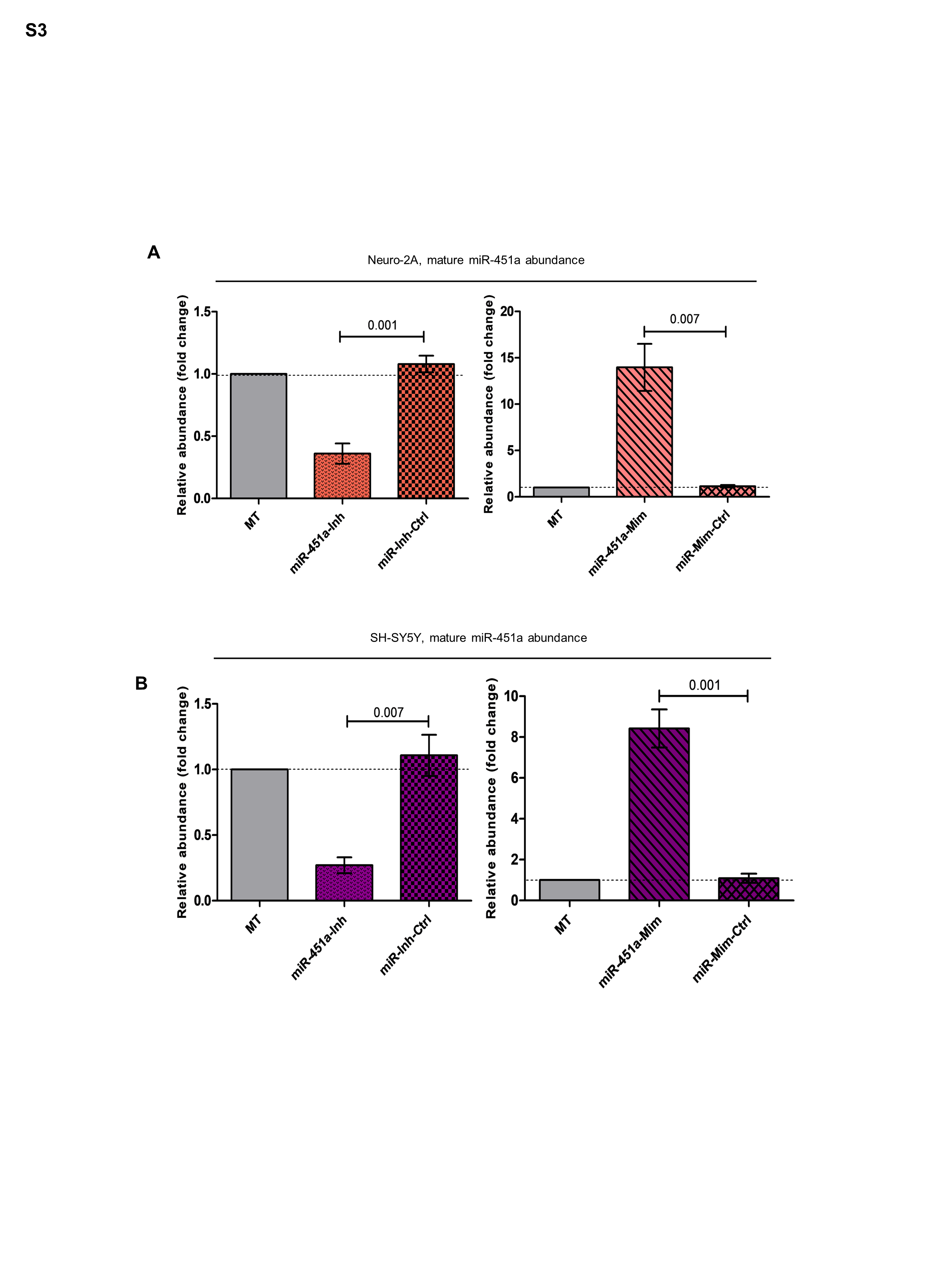

Supplement: FIG S3 [file msphere.00208-22-s0004.tif]
